# Invasive pneumococcal disease among the elderly in the later era of paediatric pneumococcal conjugate vaccination—A longitudinal study over 10 years based on public surveillance data in the Nordics

**DOI:** 10.1371/journal.pone.0287378

**Published:** 2023-06-26

**Authors:** Andreas Palmborg, Mette Skovdal, Tor Molden, Heidi Åhman, Lingjing Chen, Jonas Banefelt

**Affiliations:** 1 Pfizer Vaccines, Medical Development, Scientific and Clinical Affairs, Sweden; 2 Pfizer Vaccines, Medical Development, Scientific and Clinical Affairs, Denmark; 3 Pfizer Vaccines, Medical Development, Scientific and Clinical Affairs, Norway; 4 Pfizer Vaccines, Medical Development, Scientific and Clinical Affairs, Finland; 5 Quantify Research, Stockholm, Sweden; Universidade de Lisboa Faculdade de Medicina, PORTUGAL

## Abstract

**Background:**

Pneumococcal conjugate vaccines (PCVs) have proven effective in preventing both non-invasive and invasive pneumococcal disease (IPD) in small children and in older age groups. However, long-term observations and country comparisons of IPD incidence in the elderly following introduction of PCVs in paediatric national immunisation programmes (NIPs) are scarce. We aimed to estimate and compare incidence of IPD in the elderly in Denmark, Finland, Norway, and Sweden over a 10-year time span. During the study period Denmark and Norway used PCV13 in their paediatric NIP, Sweden both PCV10 and PCV13 and Finland used PCV10. Uptake of pneumococcal vaccines for the elderly was low.

**Method:**

We collected longitudinal data on confirmed IPD cases and their serotypes among elderly people (aged ≥65 years) 2010–2019 in the four countries of interest. Annual IPD incidence rates were calculated per country, by vaccine-associated serotypes (PCV10, PCV13, PCV15, PCV20 and PPV23) and for non-vaccine serotypes. A regression model was used to estimate average annual change in incidence in each country.

**Results:**

Incidence rates of IPD in the elderly in 2019 ranged from 31.4 to 41.8 per 100,000 people across the countries. Denmark and Norway showed an annual average decline in IPD incidence (-3.3; 95% CI: -5.6 to -1.1; p<0.01) and (-3.3; 95% CI: -5.5 to -1.0; p<0.01) respectively from 2010 to 2019, whereas no change was seen for Sweden (-0.5; 95% CI: -1.9 to 0.8; p = 0.39) or Finland (0.9; 95% CI: -1.0 to 2.7; p = 0.32). IPD incidence due to emerging serotypes, e.g., serotypes 8 and 12F, has increased. Serotype 19A remained a major cause of IPD in countries with PCV10 in paediatric NIPs.

**Conclusion:**

Despite paediatric PCV programmes, a considerable vaccine preventable IPD burden remains in the elderly. Further, choice of PCV in paediatric programs was associated with differences in serotype distribution and incidence amongst the elderly. Direct vaccination of the elderly with recently approved broad coverage PCVs holds promise for meaningful impact on disease burden with PCV20 covering a majority of IPD amongst the elderly in the four studied countries. Effectiveness of new vaccines in real-life clinical practice should be followed.

## Introduction

*Streptococcus pneumoniae* pneumococcus, is a major cause of morbidity and mortality worldwide. It affects people of all ages but small children, the elderly, and those with certain comorbidities or immunocompromising conditions are at higher risk. Pneumococcal disease is classified as either invasive pneumococcal disease (IPD) e.g., bacteraemia, bacterial meningitis and bacteraemic pneumonia or non-invasive disease, e.g., sinusitis, otitis media and non-bacteraemic pneumonia [1,2]. Non-bacteraemic pneumococcal pneumonia is the most common disease manifestation amongst adults, but the burden of IPD is also substantial, with approximately 24 000 cases reported in the European region in 2019 alone [3,4].

More than 100 serotypes of *S*. *pneumoniae* have been identified, reflecting differences in the structure of the bacterium’s polysaccharide capsule [2]. Vaccines targeting the capsular polysaccharides of prevalent serotypes are used to prevent disease with two types of vaccines available: non-conjugated pneumococcal polysaccharide vaccine (PPV) and conjugated polysaccharide vaccine (PCV) [5]. In a PCV, purified capsular polysaccharides from *S*. *pneumoniae* are covalently linked to a carrier protein to enhance immunogenicity via a T-cell–dependent immune response that promotes B-cell memory [5]. In contrast to PCV, plain non-conjugated PPVs as T-cell-independent antigens, only induce short-term immunity, do not elicit boostable memory responses, and are not effective in the elicitation of immune responses in children younger than two years [6]. The 23-valent PPV (PPV23) targets serotypes 1, 2, 3, 4, 5, 6B, 7F, 8, 9N, 9V, 10A, 11A, 12F, 14, 15B, 17F, 18C, 19F, 19A, 20, 22F, 23F and 33F. It was introduced for adults at-risk over 30 years ago, but internationally there is little evidence that PPV23 has led to any reduction in IPD, pneumococcal pneumonia, all-cause pneumonia, or their associated mortality [7,8]. The first PCV covering the 7 serotypes 4, 6B, 9V, 14, 18C, 19F and 23 (PCV7) was introduced in the early 2000s and PCVs have advanced over time since then, targeting an increasing number of specific serotypes. In Europe PCV7 (Wyeth) was licensed in 2001, followed in 2009 by PCV10 (GSK), including PCV7 serotypes plus serotypes 1, 5 and 7F, along with PCV13 (Pfizer) including PCV10 serotypes plus serotypes 3, 6A and 19A. Recently PCV15 (MSD) and PCV20 (Pfizer) have been approved for use within Europe, with PCV15 containing PCV13 serotypes plus serotypes 22F and 33F while PCV20 contains PCV15 serotypes plus serotypes 8, 10A, 11A, 12F, and 15B [9,10].

In the Nordics, Norway introduced PCV7 to its paediatric national immunisation programme (NIP) in 2006 [11], followed by Denmark in 2007 [12] and successively by Sweden for regional usage in 2007 to a national implementation in 2009 [13]. Finland introduced PCV10 in its NIP in 2010 [14]. PCV13 replaced PCV7 in the Danish and Norwegian NIPs in 2010 and 2011, respectively [11,12]. In 2010, both PCV10 and PCV13 were included in the Swedish NIP pending different regional procurement processes before switching to PCV10 only in 2019. An overview of the Nordic paediatric PCV programmes is provided in Table 1. Recommendations for pneumococcal vaccination for the elderly and those at-risk for pneumococcal disease exist but uptake up until 2019 has been limited and vaccination coverage have been estimated below 15% in all four Nordic countries [15,16]. Especially amongst the elderly the burden of disease associated with *S*. *pneumoniae* remains high [3,4] and a better understanding of pneumococcal disease patterns in the countries and the impact on the elderly due to the paediatric PCV NIPs may support improved management of disease. Our study aimed to estimate the annual incidence of IPD, in total, by vaccine-associated serotypes, and for individual serotypes, in the elderly population in Denmark, Sweden, Finland, and Norway during a 10-year time span from 2010 to 2019.

**Table 1:** PCV use in children in the Nordic countries.

Countries	Paediatric immunisation programme introduction			Paediatric PCV programme in 2019	
	PCV7	PCV10	PCV13	Schedule	Uptake
Norway [11, 16]	2006		2011	PCV13, 2+1	94%
Denmark [12, 17]	2007		2010	PCV13, 2+1	96%
Sweden [13]	2007–09	2010	2010	PCV10/PCV13, 2+1 with switch to PCV10 only in Sept 2019	97%
Finland [14]	-	2010	-	PCV10, 2+1	94%

## Material & methods

### Study population and design

This was an observational study based on public surveillance data from the four countries of interest. The total population of Denmark, Finland, Norway, and Sweden is 27 million with >5 million elderly people [17]. The annual IPD incidence rate for the period of 2010 to 2019 was estimated in the elderly aged ≥65 years, together with serotype specific coverage of licensed vaccines in the included countries.

The information on IPD incidence was obtained from the Surveillance Atlas of Infectious Disease database [18], held by the European Centre for Disease Prevention and Control (ECDC). The database contains information on different infectious diseases, collected from the European member states of ECDC through The European Surveillance System (TESSy). We accessed the annual data on the number of confirmed cases of IPD (isolation of *S*. *pneumoniae* from blood or another normally sterile site) and their associated serotypes for Denmark, Finland, Norway, and Sweden during the period of 2010 to 2019. Data on confirmed cases were available for all four countries, but complete serotype data were available only for Denmark and Finland. In Sweden, serotyped data were missing for the year 2010. Furthermore, in Norway only ~59% of IPD cases were serotyped in 2018 and 2019 [4]. For the missing serotype data, we assumed that the serotype distribution followed that of the IPD cases with available serotype information. Information on the annual population size from 2010 to 2019 for the countries was obtained from Statistics Denmark [19], Statistics Finland [20], Statistics Norway [21], and Statistics Sweden [22], respectively.

### Statistical analysis

We computed the annual incidence rate of IPD in the elderly, overall, by vaccine-associated serotypes, and for all serotypes, for each country, between 2010 and 2019. Incidence rates were calculated per 100,000 people. The vaccine serotypes included in the analyses were PCV10, PCV13, PCV15, PCV20 and PPV23.

A linear regression model was used to estimate the average annual percentage change between 2010 to 2019 in the total IPD incidence rate in the elderly as well as for individual vaccine-serotypes, in each country. Associated 95% confidence intervals (CIs) and *p*-values were generated.

All statistical analyses were performed using STATA MP version 16 (StataCorp, College Station, TX).

## Results

### Incidence rate of IPD in the elderly

**Fig 1:**
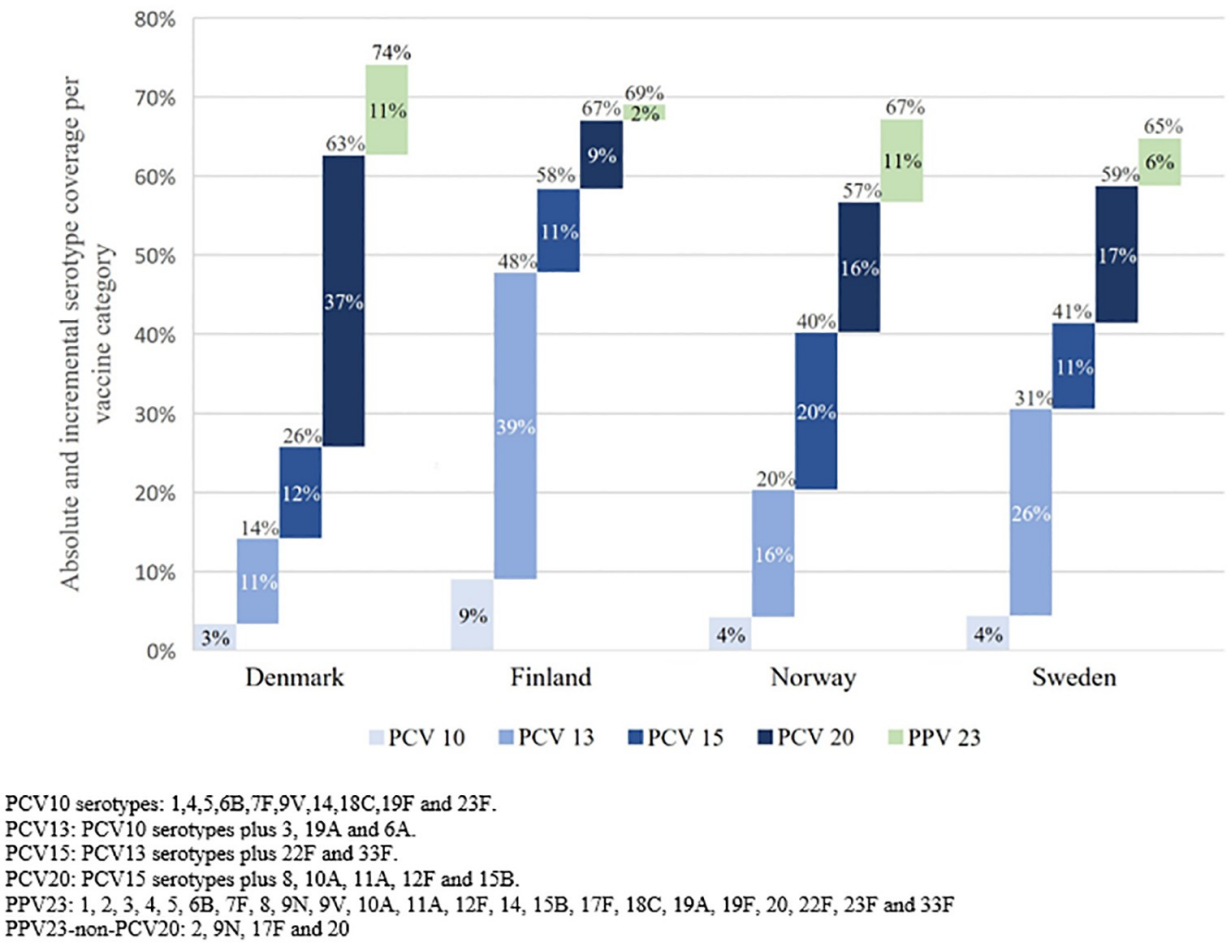
Serotype coverage of vaccine types among the elderly, average for 2017–2019.

The incidence rates of IPD in the elderly in 2019 ranged from 31.4 to 41.8 per 100,000 people across the four countries (Table 2). Sweden exhibited the highest incidence rate and Finland the lowest. The Finnish IPD incidence rate associated with PCV13 serotypes was 13.1 cases per 100,000 people, out of which 11.5 cases per 100,000 (87.8%) were associated with PCV13-non-PCV10 serotypes. Similarly, the Swedish IPD incidence rate associated with PCV13 serotypes was 12.4 per 100,000 people, out of which 10.7 cases per 100,000 (86.2%) were associated with PCV13-non-PCV10 serotypes. PCV13 serotype incidence rates for Denmark and Norway were 5.9 and 7.3 per 100,000 people, respectively. For all four countries in 2019, incidence rate of IPD associated with PCV15 serotypes ranged from 9.7 to 16.9 per 100,000 and for PCV20 between 20.2 to 24.0 per 100,000. The proportion of the total number of IPD cases associated with individual PCV serotypes, averaged between 2017–2019, is shown in Fig 1. During those years, the average proportion associated with PCV10 serotypes was 3 to 9% across the countries. For PCV15 and PCV13 serotypes, Finland exhibited the highest proportion at 58% and 48% respectively, and Denmark the lowest at 26%, and 14% respectively. For PCV20 serotypes, Finland and Denmark exhibited the highest proportion at 67 and 63%, respectively, and Norway the lowest at 57%. As such, PCV20 serotypes accounted for >50% of all IPD cases in all four countries.

**Table 2:** Incidence rates per 100,000 and average annual change of IPD among the people 65 years old and older, by vaccine serotypes, in 2010–2019 in Denmark, Finland, Norway and Sweden.

	2010	2011	2012	2013	2014	2015	2016	2017	2018	2019	Average annual change over study period (%) (95% confidence interval)	p-value
**Denmark**												
Total	54.0	52.2	50.9	43.3	42.9	48.5	41.9	42.7	46.6	35.1	-3.3 (-5.6, -1.1)	<0.01
PCV10	19.1	15.5	13.4	7.9	6.5	4.6	2.1	1.7	1.4	1.2	-34.4 (-38.4, -30.5)	<0.01
PCV13-non-PCV10	10.5	10.5	6.8	5.8	5.1	5.5	5.6	3.6	5.0	4.7	-9.6 (-14.7, -4.5)	<0.01
PCV13	29.5	26.0	20.2	13.7	11.6	10.2	7.7	5.2	6.3	5.9	-20.0 (-24.5, -15.6)	<0.01
PCV15-non-PCV13	5.2	4.6	6.7	4.8	5.4	6.1	4.5	5.0	5.7	3.9	-1.6 (-6.0, 2.8)	0.42
PCV15	34.7	30.6	27.0	18.6	16.9	16.3	12.2	10.2	12.0	9.7	-14.6 (-17.5, -11.7)	<0.01
PCV20-non-PCV15	7.9	8.8	8.4	11.0	10.8	16.0	14.2	16.7	16.1	13.0	8.1 (3.8, 12.3)	<0.01
PCV20	42.6	39.4	35.4	29.6	27.7	32.3	26.4	26.9	28.1	22.7	-5.8 (-7.7, -3.9)	0.03
PPV23	44.4	42.0	38.9	32.8	31.2	36.3	31.4	32.2	34.3	25.8	-4.4 (-6.8, -2.0)	<0.01
PPV23-non-PCV13	15.6	16.6	18.6	19.3	19.9	26.1	23.7	27.0	27.9	19.9	5.2 (0.7, 9.7)	0.03
PPV23-non-PCV20	2.6	3.2	3.5	3.5	3.7	4.0	5.0	5.3	6.2	3.1	5.7 (-2.2, 13.6)	0.14
**Finland**												
Total	32.3	30.4	33.7	30.2	32.5	38.0	36.1	37.2	32.5	31.4	0.9 (-1.0, 2.7)	0.32
PCV10	18.5	15.4	14.8	10.7	8.6	6.7	4.9	3.9	3.9	1.6	-25.0 (-31.7, -18.3)	<0.01
PCV13-non-PCV10	5.9	6.4	6.3	7.8	11.2	13.5	14.3	15.8	12.0	11.5	10.3 (4.9, 15.8)	<0.01
PCV13	24.4	21.8	21.1	18.5	19.8	20.3	19.2	19.7	15.9	13.1	-4.9 (-7.5, -2.2)	<0.01
PCV15-non-PCV13	2.5	2.8	2.7	2.7	3.9	4.5	4.9	3.7	3.2	3.8	5.1 (1.3, 8.8)	0.01
PCV15	26.8	24.6	23.8	21.1	23.7	24.7	24.1	23.4	19.1	16.9	-5.0 (-9.2, -0.9)	0.01
PCV20-non-PCV15	0.7	1.8	2.4	2.3	2.7	2.9	2.8	2.6	2.6	3.5	11.4 (0.5, 22.2)	0.04
PCV20	27.5	26.4	26.2	23.4	26.4	27.6	26.8	26.0	21.7	20.4	-2.2 (-4.2, -0.2)	0.03
PPV23	27.7	26.6	27.2	24.0	25.9	27.5	26.7	26.4	22.3	21.3	-2.1 (-3.8, -0.4)	0.02
PPV23-non-PCV13	4.8	5.9	6.7	6.5	7.8	8.3	8.4	7.6	7.1	8.3	4.7 (1.5, 7.9)	0.01
PPV23-non-PCV20	1.7	1.3	1.6	1.6	1.2	1.0	0.7	1.3	1.3	1.1	-5.0 (-9.2, -0.9)	0.02
**Norway**												
Total	54.0	51.5	42.6	38.8	38.3	37.4	44.1	37.8	38.5	38.1	-3.3 (-5.5, -1.0)	<0.01
PCV10	14.8	13.3	9.8	6.3	5.6	3.0	3.1	1.7	1.3	1.8	-28.2 (-34.3, -22.2)	<0.01
PCV13-non-PCV10	12.4	11.4	8.4	4.9	4.9	4.4	5.8	6.8	6.1	5.5	-7.5 (-13.9, -1.0)	0.03
PCV13	27.2	24.7	18.2	11.2	10.5	7.4	8.9	8.6	7.4	7.3	-15.2 (-20.1, -10.2)	<0.01
PCV15-non-PCV13	8.5	9.2	7.4	9.6	6.9	9.7	11.6	7.9	8.4	6.5	-1.1 (-4.9, 2.7)	0.52
PCV15	35.7	33.9	25.6	20.7	17.4	17.1	20.5	16.5	15.8	13.7	-9.8 (-15.5, -7.1)	<0.01
PCV20-non-PCV15	5.2	4.9	4.8	5.1	5.4	4.9	6.9	5.7	6.7	6.5	3.5 (1.7, 5.2)	<0.01
PCV20	40.9	38.8	30.4	25.8	22.8	22.0	27.3	22.2	22.5	20.2	-7.0 (-9.7, -4.4)	<0.01
PPV23	40.3	39.3	31.1	26.6	24.8	23.3	29.2	26.2	25.3	25.3	-4.8 (-7.7, -1.8)	<0.01
PPV23-non-PCV13	14.5	15.5	13.5	15.7	14.4	16.1	20.4	17.8	18.1	18.1	3.2 (1.7, 4.7)	<0.01
PPV23-non-PCV20	0.9	1.4	1.3	1.0	2.1	1.6	1.9	4.2	3.0	5.1	17.4 (11.9, 23.0)	<0.01
**Sweden**												
Total	47.0	41.1	44.3	41.6	38.6	41.1	43.7	42.9	41.9	41.8	-0.5 (-1.9, 0.8)	0.39
PCV10	N/A	11.5	9.3	5.7	5.1	3.1	2.8	2.2	1.8	1.7	-25.4 (-29.7, -21.0)	<0.01
PCV13-non-PCV10	N/A	10.3	10.3	11.4	8.7	9.8	9.6	10.3	12.0	10.7	0.8 (-1.4, 3.1)	0.41
PCV13	N/A	21.8	19.6	17.1	13.8	12.9	12.4	12.5	13.8	12.4	-6.8 (-8.7, -5.0)	<0.01
PCV15-non-PCV 13	N/A	6.7	7.1	7.0	6.6	6.1	6.3	5.4	5.0	3.5	-7.2 (-12.3, -2.0)	0.01
PCV15	N/A	28.5	26.8	24.1	20.4	19.0	18.7	17.9	18.8	15.8	-6.8 (-8.7, -5.0)	<0.01
PCV20-non-PCV 15	N/A	3.3	4.5	4.9	4.2	6.2	7.3	7.1	6.8	8.1	10.2 (6.9, 13.5)	<0.01
PCV20	N/A	31.8	31.3	29.0	24.6	25.2	26.0	24.9	25.5	24.0	-3.3 (-4.7, -1.9)	<0.01
PCV13	N/A	21.8	19.6	17.1	13.8	12.9	12.4	12.5	13.8	12.4	-6.8 (-8.7, -5.0)	<0.01
PPV23	N/A	31.1	32.6	30.1	26.7	28.0	29.0	27.2	28.1	26.6	-2.0 (-3.1, -0.8)	<0.01
PPV23-non-PCV13	N/A	11.8	13.7	14.1	13.2	15.5	16.9	15.2	14.5	14.5	2.3 (-0.2, 4.8)	0.07
PPV23-non-PCV20	N/A	1.9	2.0	2.2	2.5	3.1	3.3	2.8	2.7	2.9	5.8 (2.6, 9.1)	<0.01

### Trend of incidence rates of IPD over ten years

All four countries showed an overall decline in the incidence rate of IPD over time for the serotypes covered by the respective countries’ PCV in the NIP. The average annual decline in PCV13 serotypes related incidence rates of IPD over time was higher in Denmark (-20.0% CI: -24.5, -15.6 p<0.01) and Norway (-15.2% CI: -20.1, -10.2 p<0.01) than in Sweden (-6.8% -CI: -8.7, -5.0 p<0.01) and Finland (-4.9% CI: -7.5, -2.2 p<0.01). In Finland and Sweden, PCV10 serotypes decreased but PCV13-non-PCV10 serotypes increased in Finland and did not change in Sweden (Table 2).

Despite the overall decline over time in all four countries in incidence rates associated with the vaccine serotypes covered by each country’s respective NIP, there was no change in overall average annual incidence for Sweden (-0.5; 95% CI: -1.9 to 0.8; p = 0.39) or Finland (0.9; 95% CI: -1.0 to 2.7; p = 0.32) whereas both Denmark and Norway exhibited a significant decrease in annual IPD incidence over the study period, (-3.3; 95% CI: -5.6 to -1.1; p<0.01) and (-3.3; 95% CI: -5.5 to -1.0; p<0.01) respectively (Table 2).

### Serotype-specific analysis

Table 3 reports incidence rates per 100,000 of the most common IPD serotypes, accounting for at least 5% of the total IPD cases in the elderly in any of the countries in any one year during the study period; incidence rates for all serotypes are available in S1 Table. In 2019, the most common serotype in Denmark was 8, followed by 3, 22F, and 12F with incidence rates of 8.7 (24.9% of the total), 4.1 (11.8%), 3.2 (9.2%), and 2.2 (6.2%) respectively. Serotype 19A was the most common serotype in Finland in 2019 with 6.6 cases per 100,000 (21.1% of the total), followed by 3, 6C, and 22F with 4.7 (15.1%), 4.2 (13.3%), and 3.6 (11.5%) respectively. In Norway, the most common serotype in 2019 was 22F followed by 3, 9N, and 8 with 5.5 (14.4% of the total), 5.1 (13.4%), 3.9 (10.3%), and 3.3 (8.8%) IPD cases per 100,000, respectively. Serotype 3 was the most common serotype in Sweden in 2019 with 6.4 (15.4% of the total) IPD cases per 100,000. Other common serotypes in Sweden included 19A, 8, 15A, 22F and 6C with 4.0 (9.7%), 3.8 (9.0%), 3.0 (7.2%), 2.8 (6.6%) and 2.6 (6.1%) of cases per 100,000, respectively.

**Table 3:** Incidence rates per 100,000 of common* IPD serotypes among elderly 2010–2019, by vaccine serotypes, in Denmark, Finland, Norway, and Sweden, sorted by frequency in 2019 per country. Parentheses show the percentage of the total (%).

**Denmark**	**2010**	**2011**	**2012**	**2013**	**2014**	**2015**	**2016**	**2017**	**2018**	**2019**
**Serotype**										
**Total**	**54.0 (100)**	**52.2 (100)**	**50.9 (100)**	**43.3 (100)**	**42.9 (100)**	**48.5 (100)**	**41.9 (100)**	**42.7 (100)**	**46.6 (100)**	**35.1 (100)**
8	3.3 (6.2)	3.8 (7.2)	3.2 (6.2)	5.0 (11.6)	7.1 (16.5)	10.5 (21.6)	9.1 (21.7)	10.2 (23.9)	10.5 (22.5)	8.7 (24.9)
3	4.6 (8.6)	5.5 (10.6)	4.6 (9.0)	3.7 (8.4)	3.5 (8.2)	4.5 (9.4)	4.3 (10.4)	3.3 (7.7)	4.5 (9.7)	4.1 (11.8)
22F	4.0 (7.4)	3.2 (6.2)	4.8 (9.4)	3.8 (8.7)	3.1 (7.1)	4.4 (9.2)	3.3 (7.9)	4.2 (9.9)	4.6 (9.9)	3.2 (9.2)
12F	1.4 (2.6)	2.6 (5.0)	2.3 (4.6)	3.4 (7.8)	1.8 (4.2)	2.5 (5.1)	2.5 (5.9)	3.7 (8.6)	2.6 (5.5)	2.2 (6.2)
9N	1.8 (3.4)	2.2 (4.2)	3.0 (5.8)	2.5 (5.7)	2.8 (6.5)	3.1 (6.3)	2.8 (6.8)	3.3 (7.7)	3.9 (8.5)	1.8 (5.1)
15A	0.2 (0.4)	0.8 (1.6)	1.3 (2.6)	2.0 (4.6)	2.1 (4.9)	2.1 (4.3)	1.4 (3.4)	0.8 (2.0)	1.6 (3.4)	1.2 (3.3)
23A	1.2 (2.2)	0.9 (1.8)	0.4 (0.8)	1.1 (2.5)	0.9 (2.0)	0.3 (0.6)	0.9 (2.3)	1.3 (3.1)	0.9 (2.0)	0.9 (2.6)
23B	0.4 (0.8)	0.8 (1.6)	0.9 (1.8)	0.7 (1.6)	0.5 (1.1)	0.9 (1.8)	1.0 (2.5)	0.9 (2.2)	0.8 (1.8)	0.7 (2.1)
24F	0.8 (1.4)	0.6 (1.2)	2.0 (4.0)	0.8 (1.8)	2.6 (6.0)	2.0 (4.1)	1.4 (3.4)	0.8 (2.0)	1.1 (2.4)	0.7 (2.1)
33F	1.2 (2.2)	1.4 (2.6)	1.9 (3.8)	1.1 (2.5)	2.3 (5.3)	1.7 (3.5)	1.2 (2.9)	0.7 (1.8)	1.1 (2.4)	0.6 (1.8)
10A	1.3 (2.4)	0.7 (1.4)	1.4 (2.8)	0.7 (1.6)	0.7 (1.6)	0.9 (1.8)	1.4 (3.4)	0.7 (1.5)	1.0 (2.2)	0.5 (1.5)
19A	5.1 (9.4)	4.4 (8.4)	2.2 (4.4)	2.0 (4.6)	1.3 (3.1)	1.0 (2.0)	1.2 (2.9)	0.3 (0.7)	0.5 (1.0)	0.5 (1.5)
19F	1.3 (2.4)	0.9 (1.8)	0.6 (1.2)	0.6 (1.4)	0.6 (1.3)	1.1 (2.2)	0.5 (1.1)	0.8 (2.0)	0.5 (1.0)	0.5 (1.5)
6C	1.6 (3.0)	2.4 (4.6)	1.8 (3.6)	0.7 (1.6)	0.7 (1.6)	1.2 (2.4)	0.8 (1.8)	0.7 (1.8)	0.4 (0.8)	0.4 (1.0)
4	2.2 (4.0)	1.3 (2.4)	1.1 (2.2)	0.7 (1.6)	1.0 (2.2)	0.4 (0.8)	0.3 (0.7)	0.0 (0.0)	0.4 (0.8)	0.3 (0.8)
7F	4.5 (8.4)	3.5 (6.8)	3.5 (6.8)	2.1 (4.8)	2.8 (6.5)	1.8 (3.7)	0.6 (1.4)	0.4 (0.9)	0.4 (0.8)	0.2 (0.5)
14	1.4 (2.6)	0.7 (1.4)	0.6 (1.2)	0.2 (0.5)	0.0 (0.0)	0.2 (0.4)	0.0 (0.0)	0.2 (0.4)	0.2 (0.4)	0.1 (0.3)
1	6.6 (12.2)	7.3 (14.0)	6.7 (13.2)	4.1 (9.4)	1.6 (3.8)	0.8 (1.6)	0.1 (0.2)	0.0 (0.0)	0.0 (0.0)	0.0 (0.0)
6A	0.8 (1.4)	0.6 (1.2)	0.0 (0.0)	0.2 (0.5)	0.2 (0.4)	0.0 (0.0)	0.0 (0.0)	0.0 (0.0)	0.0 (0.0)	0.0 (0.0)
6B	0.5 (1.0)	0.5 (1.0)	0.3 (0.6)	0.0 (0.0)	0.4 (0.9)	0.1 (0.2)	0.3 (0.7)	0.0 (0.0)	0.0 (0.0)	0.0 (0.0)
23F	0.6 (1.2)	0.4 (0.8)	0.2 (0.4)	0.2 (0.5)	0.1 (0.2)	0.1 (0.2)	0.3 (0.7)	0.0 (0.0)	0.0 (0.0)	0.0 (0.0)
**Finland**	**2010**	**2011**	**2012**	**2013**	**2014**	**2015**	**2016**	**2017**	**2018**	**2019**
**Serotype**										
**Total**	**32.3 (100)**	**30.4 (100)**	**33.7 (100)**	**30.2 (100)**	**32.5 (100)**	**38.0 (100)**	**36.1 (100)**	**37.2 (100)**	**32.5 (100)**	**31.4 (100)**
19A	1.1 (3.4)	1.7 (5.4)	2.1 (6.2)	3.3 (11.0)	3.7 (11.4)	5.5 (14.4)	6.9 (19.1)	8.5 (22.9)	7.1 (21.7)	6.6 (21.1)
3	3.3 (10.3)	3.6 (11.9)	3.6 (10.7)	3.5 (11.6)	5.8 (17.7)	7.0 (18.4)	6.5 (18.1)	6.4 (17.3)	4.3 (13.2)	4.7 (15.1)
6C	0.1 (0.3)	0.7 (2.4)	1.5 (4.5)	1.2 (4.1)	0.7 (2.3)	2.7 (7.1)	2.8 (7.7)	3.8 (10.2)	3.4 (10.6)	4.2 (13.3)
22F	2.5 (7.6)	2.8 (9.2)	2.7 (8.0)	2.7 (8.8)	3.9 (12.0)	4.0 (10.6)	4.2 (11.6)	3.4 (9.2)	2.7 (8.3)	3.6 (11.5)
15A	0.2 (0.7)	0.2 (0.7)	0.3 (0.9)	0.3 (0.9)	0.2 (0.6)	0.5 (1.4)	1.0 (2.7)	0.4 (1.2)	0.4 (1.3)	1.1 (3.6)
23B	0.1 (0.3)	0.0 (0.0)	0.1 (0.3)	0.1 (0.3)	0.1 (0.3)	0.9 (2.4)	0.2 (0.5)	1.2 (3.2)	1.5 (4.7)	1.1 (3.4)
8	0.0 (0.0)	0.2 (0.7)	0.3 (0.9)	0.1 (0.3)	0.4 (1.1)	0.1 (0.2)	1.0 (2.9)	0.9 (2.3)	0.6 (1.8)	1.0 (3.1)
9N	1.7 (5.2)	1.2 (4.1)	1.5 (4.5)	1.5 (5.0)	0.9 (2.9)	1.0 (2.6)	0.6 (1.7)	0.9 (2.3)	0.6 (1.8)	0.8 (2.6)
23A	0.3 (1.0)	0.3 (1.0)	0.8 (2.4)	0.6 (1.9)	0.8 (2.6)	1.9 (5.0)	1.6 (4.3)	1.3 (3.5)	1.2 (3.6)	0.7 (2.3)
19F	2.3 (7.2)	0.9 (3.1)	1.5 (4.5)	0.8 (2.5)	0.5 (1.4)	0.6 (1.7)	0.8 (2.2)	0.1 (0.2)	0.4 (1.3)	0.6 (1.8)
4	2.1 (6.6)	1.6 (5.1)	1.5 (4.5)	1.8 (6.0)	1.1 (3.4)	1.3 (3.5)	1.2 (3.4)	0.9 (2.3)	1.1 (3.4)	0.3 (1.0)
6A	1.4 (4.5)	1.1 (3.7)	0.6 (1.8)	0.9 (3.1)	1.7 (5.1)	1.1 (2.8)	0.9 (2.4)	0.9 (2.3)	0.7 (2.1)	0.2 (0.5)
6B	2.7 (8.3)	2.0 (6.5)	1.6 (4.7)	0.9 (2.8)	0.5 (1.4)	1.2 (3.1)	0.4 (1.2)	0.5 (1.4)	0.4 (1.3)	0.2 (0.8)
10A	0.0 (0.0)	0.2 (0.7)	0.2 (0.6)	0.1 (0.3)	0.3 (0.9)	0.3 (0.7)	0.2 (0.5)	0.2 (0.5)	0.5 (1.6)	0.2 (0.8)
12F	0.0 (0.0)	0.0 (0.0)	0.1 (0.3)	0.1 (0.3)	0.3 (0.9)	0.2 (0.5)	0.1 (0.2)	0.1 (0.2)	0.0 (0.0)	0.2 (0.5)
24F	0.0 (0.0)	0.0 (0.0)	0.0 (0.0)	0.0 (0.0)	0.0 (0.0)	0.3 (0.7)	0.3 (0.7)	0.2 (0.5)	0.3 (0.8)	0.2 (0.8)
33F	0.0 (0.0)	0.0 (0.0)	0.0 (0.0)	0.0 (0.0)	0.0 (0.0)	0.4 (1.2)	0.7 (1.9)	0.3 (0.7)	0.5 (1.6)	0.2 (0.5)
14	5.2 (16.2)	4.7 (15.3)	4.8 (14.2)	2.8 (9.4)	2.1 (6.6)	0.4 (1.2)	0.7 (1.9)	0.9 (2.3)	0.5 (1.6)	0.1 (0.3)
23F	2.8 (8.6)	2.3 (7.5)	2.5 (7.4)	2.2 (7.2)	2.1 (6.6)	1.4 (3.8)	0.7 (1.9)	0.8 (2.1)	0.3 (1.0)	0.1 (0.3)
1	0.0 (0.0)	0.0 (0.0)	0.0 (0.0)	0.0 (0.0)	0.1 (0.3)	0.1 (0.2)	0.0 (0.0)	0.0 (0.0)	0.0 (0.0)	0.0 (0.0)
7F	1.6 (4.8)	1.6 (5.1)	1.1 (3.3)	0.8 (2.5)	0.8 (2.6)	0.5 (1.4)	0.3 (1.0)	0.1 (0.2)	0.4 (1.3)	0.0 (0.0)
**Norway**	**2010**	**2011**	**2012**	**2013**	**2014**	**2015**	**2016**	**2017**	**2018**	**2019**
**Serotype**										
**Total**	**54.0 (100)**	**51.5 (100)**	**42.6 (100)**	**38.8 (100)**	**38.3 (100)**	**37.4 (100)**	**44.1 (100)**	**37.8 (100)**	**38.5 (100)**	**38.1 (100)**
22F	6.2 (11.5)	6.5 (12.5)	6.2 (14.5)	7.7 (19.9)	6.1 (16.1)	7.8 (20.8)	9.3 (21.0)	6.6 (17.5)	7.0 (18.3)	5.5 (14.4)
3	4.2 (7.7)	3.4 (6.7)	4.0 (9.4)	2.6 (6.8)	2.6 (6.9)	2.7 (7.1)	3.9 (8.7)	5.9 (15.6)	4.8 (12.4)	5.1 (13.4)
9N	0.6 (1.1)	1.2 (2.4)	1.1 (2.5)	0.8 (2.0)	1.9 (4.9)	1.5 (3.9)	1.6 (3.6)	3.1 (8.3)	2.5 (6.4)	3.9 (10.3)
8	1.2 (2.1)	1.2 (2.4)	1.1 (2.5)	0.9 (2.4)	1.4 (3.6)	1.2 (3.2)	2.0 (4.6)	2.3 (6.1)	2.1 (5.4)	3.3 (8.8)
23B	0.3 (0.5)	1.2 (2.4)	2.0 (4.7)	2.5 (6.4)	1.5 (3.9)	1.8 (4.9)	1.0 (2.2)	1.4 (3.7)	1.5 (4.0)	2.2 (5.7)
23A	2.3 (4.3)	2.2 (4.3)	1.2 (2.8)	1.6 (4.1)	2.3 (5.9)	1.7 (4.5)	0.7 (1.6)	2.1 (5.5)	1.1 (3.0)	1.6 (4.1)
10A	1.2 (2.1)	0.5 (1.1)	0.9 (2.2)	1.2 (3.0)	1.0 (2.6)	1.2 (3.2)	1.3 (3.0)	1.3 (3.4)	2.1 (5.4)	1.4 (3.6)
15A	0.0 (0.0)	0.1 (0.3)	0.8 (1.9)	0.5 (1.4)	1.3 (3.3)	1.1 (2.9)	1.6 (3.6)	1.9 (4.9)	1.9 (5.0)	1.4 (3.6)
6C	3.6 (6.7)	4.0 (7.7)	2.7 (6.3)	2.5 (6.4)	1.5 (3.9)	1.5 (3.9)	1.0 (2.2)	0.3 (0.9)	1.7 (4.5)	1.0 (2.6)
24F	0.1 (0.3)	0.1 (0.3)	0.3 (0.6)	0.3 (0.7)	0.8 (2.0)	1.7 (4.5)	2.2 (4.9)	1.4 (3.7)	0.2 (0.5)	1.0 (2.6)
33F	2.3 (4.3)	2.7 (5.3)	1.2 (2.8)	1.8 (4.7)	0.8 (2.0)	1.9 (5.2)	2.3 (5.2)	1.3 (3.4)	1.3 (3.5)	1.0 (2.6)
19F	1.2 (2.1)	1.1 (2.1)	0.8 (1.9)	0.4 (1.0)	0.3 (0.7)	0.5 (1.3)	0.6 (1.4)	0.3 (0.9)	0.4 (1.0)	0.8 (2.1)
14	1.4 (2.7)	0.7 (1.3)	0.5 (1.3)	0.4 (1.0)	0.3 (0.7)	0.2 (0.6)	0.2 (0.5)	0.1 (0.3)	0.0 (0.0)	0.4 (1.0)
19A	6.8 (12.5)	7.0 (13.6)	3.7 (8.8)	2.0 (5.1)	2.1 (5.6)	1.5 (3.9)	1.8 (4.1)	0.8 (2.1)	1.1 (3.0)	0.4 (1.0)
4	2.2 (4.0)	1.2 (2.4)	1.1 (2.5)	0.8 (2.0)	0.1 (0.3)	0.2 (0.6)	0.4 (0.8)	0.1 (0.3)	0.2 (0.5)	0.2 (0.5)
7F	5.3 (9.9)	5.4 (10.4)	4.7 (11.0)	3.3 (8.4)	3.1 (8.2)	1.7 (4.5)	1.7 (3.8)	0.3 (0.9)	0.4 (1.0)	0.2 (0.5)
1	2.0 (3.7)	2.1 (4.0)	0.9 (2.2)	0.4 (1.0)	0.5 (1.3)	0.0 (0.0)	0.0 (0.0)	0.0 (0.0)	0.0 (0.0)	0.0 (0.0)
6A	1.4 (2.7)	1.0 (1.9)	0.7 (1.6)	0.3 (0.7)	0.1 (0.3)	0.2 (0.6)	0.1 (0.3)	0.1 (0.3)	0.2 (0.5)	0.0 (0.0)
6B	0.0 (0.0)	1.1 (2.1)	0.7 (1.6)	0.4 (1.0)	0.5 (1.3)	0.1 (0.3)	0.0 (0.0)	0.2 (0.6)	0.4 (1.0)	0.0 (0.0)
12F	0.1 (0.3)	0.5 (1.1)	0.3 (0.6)	0.5 (1.4)	0.5 (1.3)	0.4 (1.0)	1.2 (2.7)	0.5 (1.2)	0.6 (1.5)	0.0 (0.0)
23F	0.7 (1.3)	0.8 (1.6)	0.3 (0.6)	0.4 (1.0)	0.1 (0.3)	0.0 (0.0)	0.0 (0.0)	0.2 (0.6)	0.0 (0.0)	0.0 (0.0)
**Sweden**	**2010**	**2011**	**2012**	**2013**	**2014**	**2015**	**2016**	**2017**	**2018**	**2019**
**Serotype**										
**Total**	**47.0 (100)**	**41.1 (100)**	**44.3 (100)**	**41.6 (100)**	**38.6 (100)**	**41.1 (100)**	**43.7 (100)**	**42.9 (100)**	**41.9 (100)**	**41.8 (100)**
3	N/A	4.0 (9.7)	6.5 (14.6)	7.1 (17.0)	5.5 (14.3)	5.7 (13.9)	6.8 (15.5)	6.1 (14.3)	7.3 (17.3)	6.4 (15.4)
19A	N/A	3.8 (9.2)	3.2 (7.2)	3.2 (7.8)	2.9 (7.5)	3.7 (8.9)	2.5 (5.7)	3.7 (8.6)	4.6 (11.1)	4.0 (9.7)
8	N/A	0.7 (1.7)	0.8 (1.7)	0.8 (1.8)	0.9 (2.3)	1.8 (4.3)	2.1 (4.7)	2.8 (6.5)	3.7 (8.7)	3.8 (9.0)
15A	N/A	0.1 (0.1)	0.3 (0.7)	0.7 (1.6)	0.6 (1.6)	0.9 (2.1)	1.8 (4.1)	2.0 (4.7)	2.4 (5.8)	3.0 (7.2)
22F	N/A	5.0 (12.2)	5.3 (12.0)	5.1 (12.2)	4.8 (12.3)	4.2 (10.1)	4.8 (11.0)	4.6 (10.7)	4.3 (10.2)	2.8 (6.6)
6C	N/A	2.0 (4.9)	1.8 (4.1)	2.7 (6.5)	2.5 (6.4)	2.9 (7.1)	2.7 (6.1)	2.7 (6.4)	2.6 (6.2)	2.6 (6.1)
9N	N/A	1.6 (3.9)	1.8 (4.1)	1.9 (4.6)	2.2 (5.8)	2.6 (6.4)	3.0 (6.9)	2.4 (5.5)	1.9 (4.6)	2.1 (5.0)
23A	N/A	1.5 (3.6)	2.1 (4.7)	1.2 (2.8)	2.1 (5.4)	1.2 (3.0)	1.1 (2.4)	2.0 (4.6)	2.0 (4.8)	1.8 (4.4)
23B	N/A	0.1 (0.3)	0.4 (1.0)	0.9 (2.1)	1.6 (4.1)	1.8 (4.3)	1.5 (3.5)	1.9 (4.4)	1.1 (2.7)	1.4 (3.4)
10A	N/A	0.7 (1.6)	0.8 (1.7)	0.9 (2.1)	1.1 (3.0)	1.8 (4.3)	1.3 (3.0)	1.1 (2.5)	1.0 (2.5)	0.9 (2.3)
12F	N/A	0.2 (0.4)	0.3 (0.6)	0.4 (0.9)	0.4 (1.1)	0.5 (1.3)	1.6 (3.6)	0.9 (2.0)	0.9 (2.2)	0.8 (2.0)
33F	N/A	1.7 (4.1)	1.8 (4.1)	2.0 (4.8)	1.8 (4.7)	2.0 (4.8)	1.5 (3.4)	0.8 (1.8)	0.7 (1.7)	0.7 (1.6)
24F	N/A	0.2 (0.4)	0.3 (0.6)	0.3 (0.7)	0.5 (1.3)	1.0 (2.4)	2.2 (4.9)	1.5 (3.5)	1.1 (2.6)	0.6 (1.4)
7F	N/A	2.0 (4.9)	2.1 (4.8)	1.5 (3.7)	1.8 (4.7)	0.9 (2.2)	0.6 (1.4)	0.3 (0.7)	0.3 (0.6)	0.5 (1.1)
4	N/A	1.0 (2.5)	0.8 (1.8)	0.8 (1.8)	0.3 (0.7)	0.3 (0.7)	0.2 (0.4)	0.3 (0.7)	0.3 (0.6)	0.2 (0.5)
6A	N/A	2.6 (6.3)	0.6 (1.4)	1.1 (2.6)	0.3 (0.8)	0.4 (0.9)	0.3 (0.7)	0.5 (1.2)	0.1 (0.2)	0.2 (0.5)
6B	N/A	1.3 (3.1)	0.9 (2.0)	0.7 (1.7)	0.8 (2.1)	0.4 (0.9)	0.2 (0.4)	0.5 (1.2)	0.3 (0.6)	0.2 (0.5)
14	N/A	1.9 (4.5)	1.1 (2.4)	0.4 (0.9)	0.7 (1.7)	0.4 (0.9)	0.1 (0.1)	0.2 (0.5)	0.2 (0.5)	0.2 (0.4)
19F	N/A	0.6 (1.5)	0.9 (2.1)	0.5 (1.2)	0.4 (1.1)	0.3 (0.7)	1.1 (2.5)	0.3 (0.7)	0.5 (1.1)	0.2 (0.4)
1	N/A	0.2 (0.6)	0.5 (1.1)	0.2 (0.5)	0.1 (0.3)	0.1 (0.1)	0.1 (0.1)	0.1 (0.2)	0.0 (0.0)	0.1 (0.1)
23F	N/A	2.2 (5.4)	1.3 (3.0)	0.5 (1.2)	0.4 (1.0)	0.2 (0.4)	0.1 (0.2)	0.2 (0.5)	0.1 (0.2)	0.1 (0.3)

*The table shows serotypes accounting for at least 5% of the total number of IPD cases in any country for at least one year during the study period.

## Discussion

In this longitudinal observational study, we present data from 10 years of national immunisation programmes in Denmark, Finland, Norway, and Sweden. We evaluated the trend of indirect impact of paediatric NIPs in the elderly, defined as those aged 65 years and above, in the later era of paediatric pneumococcal conjugate vaccination. As the Nordic countries are similar in terms of demographics and health care system, the country comparison becomes relevant and variances observed in IPD serotype distribution in the 65+ population can likely be attributed to differences in paediatric PCV immunization, in particular the choice between PCV10 or PCV13, especially considering the low vaccine uptake in the elderly and at-risk adults during the time period under study [15].

Firstly, across the Nordic regions IPD caused by vaccine preventable serotypes are still relatively common, despite robust paediatric PCV immunization. This finding is in line with experiences from several European countries [15,23] and the observation emphasizes the limits of relying on paediatric pneumococcal vaccination solely to also manage disease amongst the elderly. Moreover, if other non-invasive disease manifestations caused by the bacterium e.g., pneumococcal pneumonia were to be considered, the overall vaccine preventable disease burden would multiply several folds [3]. In recent years, pneumococcal conjugate vaccine efficacy and effectiveness against both IPD and community acquired pneumonia caused by vaccine serotypes have been documented amongst adults following vaccination [24,25], as well as impact on all-cause pneumonia and lower respiratory infections following direct conjugate vaccination of adults [26–28]. In contrast, robust data for PPV23 vaccination demonstrating meaningful impact on disease burden is still scarce despite longstanding use [7,8].

Secondly, choice of PCV in the pediatric program was associated with differences in disease burden amongst the elderly, in terms of both disease incidence and serotype distribution. Of particular note is a decline amongst elderly for Denmark and Norway using PCV13 in overall IPD incidence, corresponding to a change in total incidence between the years 2010 and 2019 of -35.0% and -29.4% respectively whereas no change was seen in Sweden or Finland. For Denmark and Norway, a decline in IPD incidence across all ages including the elderly following pediatric PCV vaccination have previously been documented [11,12]. For Sweden, decreases in pediatric and adult IPD incidences but not amongst the elderly have been previously described [13], an observation similar to what has been noted for Finland [29]. Also, for Denmark and Norway versus Finland using PCV10 a different serotype distribution can also be noted: amongst PCV13-non-PCV10 serotypes (19A, 6A and 3) 19A was the first most common serotype in Finland amongst elderly in 2019 (21% of cases) whereas only present at very low levels in Denmark and Norway. In Sweden (usage of both PCV10 and PCV13) serotype 19A was second most common (10% of cases). Across the Nordic countries only low levels of 6A could be seen, but serotype 6C which is described as cross-reactive with 6A [30] appeared only at a very low proportions in Denmark and Norway, 1% and 3% respectively whereas more common especially in Finland with 13% of cases. In our study serotype 3 remained a common cause of IPD amongst elderly in all four countries but somewhat more common in Finland and Sweden during the study period. PCV13 protection against serotype 3 have in literature been discussed and no vaccine effectiveness against IPD has been reported [31], as well as limited effect on serotype 3 carriage [30]. However, later evidence supports PCV13 effectiveness in preventing serotype 3 pneumococcal disease; this includes both observational data as well as serotype specific analysis of data from randomized clinical trial in directly vaccinated adults [24–26], effectiveness against serotype 3 IPD in European children [32] and serotype specific effectiveness in AOM [33]. Direct vaccination, i.e., improving vaccination coverage, of the elderly is therefore a potential strategy to protect against serotype 3 disease besides pediatric vaccination with a serotype 3 containing vaccine.

Thirdly, PCV20 provides the broadest serotype coverage amongst PCVs and PCV20 serotypes accounted for a majority of IPD amongst the elderly in all four studied countries in our comparison. This warrants considerations to the benefits of the conjugated vaccine (T–cell–dependent immunity, antibody response, efficacy/effectiveness against IPD and non-bacteremic disease, length of protection) [24,25,34] when deciding upon which vaccine to use for vaccinating the elderly. Revised guidelines in the US now recommend either PCV20 as single vaccination or PCV15 followed by a dose of PPV23 when vaccinating those ≥65 years of age and at-risk adults at 18–64 years of age [35]. In addition, the choice of PCV in paediatric programs warrants careful consideration and the need for conjugated vaccines providing broad serotype coverage for both children and adults in Europe have recently been articulated [15].

## Limitations

Our study, being a surveillance database-based study with confirmed IPD cases and associated serotypes for a specific country and year, did not include any adjustments for these or any other potential confounding factor. Neither were biases possibly attributed to surveillance methodologies, other health interventions or secular trends of individual serotypes accounted for. Of note in our study is that both Denmark and Norway started from a higher incidence of IPD amongst elderly than both Finland and Sweden. Also, Finland, although no noted decline in incidence during the study years, remained at a lower overall IPD incidence suggesting other differences exists in-between countries e.g., differences in sampling, surveillance and reporting practices; in Finland under-ascertainment of IPD cases have previously been described [36]. The analysis is further complicated by the fact that Sweden used both PCV10 and PCV13 during the study period whereas its neighbouring countries used either PCV10 or PCV13. The more relevant comparison of PCV10 and PCV13 differential impact is therefore Denmark and Norway (PCV13) versus Finland (PCV10), with Sweden being somewhat of an intermediate in-between.

## Conclusion

Despite paediatric PCV programmes, there remains a considerable preventable IPD burden in the elderly. Also, choice of PCV in paediatric programs is associated with differences in serotype distribution and disease incidence amongst older adults. Direct vaccination of the elderly with recently approved broad coverage conjugate vaccines holds promise for meaningful impact on disease burden with PCV20 covering a majority of IPD amongst the elderly in all Nordic countries. Effectiveness of new vaccines in real-life clinical practice should be followed.

## Supporting information

S1 Table Incidence rates per 100,000 of each IPD serotype among elderly 2010–2019, in Denmark, Finland, Norway, and Sweden, sorted alphabetically. (DOCX)
