# Supplementary material for: Invasive pneumococcal disease among the elderly in the later era of paediatric pneumococcal conjugate vaccination—A longitudinal study over 10 years based on public surveillance data in the Nordics
Source: PLoS One. 2023 Jun 26;18(6):e0287378. doi: 10.1371/journal.pone.0287378 (PMC10292715; doi:10.1371/journal.pone.0287378)
Supplement: S1 Table — (DOCX) [file pone.0287378.s001.docx]

**S1 Table. Incidence rates per 100,000 of each IPD serotype among elderly 2010-2019, in Denmark, Finland, Norway, and Sweden, sorted alphabetically**

| **Denmark** | **2010** | **2011** | **2012** | **2013** | **2014** | **2015** | **2016** | **2017** | **2018** | **2019** |
| --- | --- | --- | --- | --- | --- | --- | --- | --- | --- | --- |
| **Serotype** |  | | | | | | | | | |
| **Total** | **54.0** | **52.2** | **50.9** | **43.3** | **42.9** | **48.5** | **41.9** | **42.7** | **46.6** | **35.1** |
| 1 | 6.6 | 7.3 | 6.7 | 4.1 | 1.6 | 0.8 | 0.1 | 0.0 | 0.0 | 0.0 |
| 10 | 0.0 | 0.0 | 0.0 | 0.0 | 0.0 | 0.0 | 0.0 | 0.0 | 0.0 | 0.0 |
| 10A | 1.3 | 0.7 | 1.4 | 0.7 | 0.7 | 0.9 | 1.4 | 0.7 | 1.0 | 0.5 |
| 10B | 0.2 | 0.4 | 0.4 | 0.8 | 0.2 | 0.5 | 0.4 | 0.3 | 0.6 | 0.4 |
| 10C | 0.0 | 0.0 | 0.0 | 0.0 | 0.0 | 0.0 | 0.0 | 0.0 | 0.0 | 0.0 |
| 10F | 0.0 | 0.0 | 0.0 | 0.0 | 0.0 | 0.0 | 0.0 | 0.0 | 0.0 | 0.0 |
| 11 | 0.0 | 0.0 | 0.0 | 0.0 | 0.0 | 0.0 | 0.0 | 0.0 | 0.0 | 0.0 |
| 11A | 1.4 | 1.4 | 1.2 | 1.4 | 1.1 | 1.6 | 0.8 | 1.4 | 1.4 | 1.1 |
| 11B | 0.0 | 0.0 | 0.0 | 0.0 | 0.0 | 0.1 | 0.0 | 0.0 | 0.1 | 0.0 |
| 11C | 0.0 | 0.0 | 0.0 | 0.0 | 0.0 | 0.0 | 0.0 | 0.0 | 0.1 | 0.0 |
| 11D | 0.0 | 0.0 | 0.0 | 0.0 | 0.0 | 0.0 | 0.0 | 0.0 | 0.0 | 0.0 |
| 11E | 0.0 | 0.0 | 0.0 | 0.0 | 0.0 | 0.0 | 0.0 | 0.0 | 0.0 | 0.0 |
| 11F | 0.0 | 0.0 | 0.0 | 0.0 | 0.0 | 0.0 | 0.0 | 0.0 | 0.0 | 0.0 |
| 12 | 0.0 | 0.0 | 0.0 | 0.0 | 0.0 | 0.2 | 0.0 | 0.0 | 0.0 | 0.0 |
| 12A | 0.0 | 0.0 | 0.0 | 0.0 | 0.0 | 0.0 | 0.0 | 0.0 | 0.0 | 0.0 |
| 12B | 0.0 | 0.0 | 0.0 | 0.0 | 0.0 | 0.0 | 0.0 | 0.0 | 0.0 | 0.0 |
| 12F | 1.4 | 2.6 | 2.3 | 3.4 | 1.8 | 2.5 | 2.5 | 3.7 | 2.6 | 2.2 |
| 13 | 0.0 | 0.0 | 0.0 | 0.0 | 0.0 | 0.0 | 0.0 | 0.0 | 0.0 | 0.0 |
| 14 | 1.4 | 0.7 | 0.6 | 0.2 | 0.0 | 0.2 | 0.0 | 0.2 | 0.2 | 0.1 |
| 15 | 0.0 | 0.0 | 0.0 | 0.0 | 0.0 | 0.2 | 0.0 | 0.0 | 0.0 | 0.0 |
| 15A | 0.2 | 0.8 | 1.3 | 2.0 | 2.1 | 2.1 | 1.4 | 0.8 | 1.6 | 1.2 |
| 15B | 0.4 | 0.3 | 0.3 | 0.5 | 0.2 | 0.6 | 0.5 | 0.7 | 0.6 | 0.5 |
| 15B/C | 0.0 | 0.0 | 0.0 | 0.0 | 0.0 | 0.0 | 0.0 | 0.0 | 0.0 | 0.0 |
| 15C | 0.8 | 0.3 | 0.4 | 0.5 | 0.2 | 0.3 | 0.9 | 0.2 | 0.2 | 0.5 |
| 15F | 0.0 | 0.0 | 0.1 | 0.0 | 0.0 | 0.0 | 0.0 | 0.0 | 0.0 | 0.0 |
| 16 | 0.0 | 0.0 | 0.0 | 0.0 | 0.0 | 0.0 | 0.0 | 0.0 | 0.0 | 0.0 |
| 16A | 0.0 | 0.0 | 0.0 | 0.0 | 0.0 | 0.0 | 0.0 | 0.0 | 0.0 | 0.0 |
| 16F | 0.6 | 0.4 | 0.7 | 0.5 | 0.8 | 1.0 | 0.9 | 1.5 | 1.6 | 1.3 |
| 17 | 0.0 | 0.0 | 0.0 | 0.0 | 0.0 | 0.0 | 0.0 | 0.0 | 0.0 | 0.0 |
| 17A | 0.0 | 0.1 | 0.0 | 0.0 | 0.0 | 0.1 | 0.1 | 0.1 | 0.3 | 0.3 |
| 17F | 0.0 | 0.4 | 0.0 | 0.2 | 0.1 | 0.1 | 0.7 | 0.6 | 0.7 | 0.5 |
| 18 | 0.0 | 0.0 | 0.0 | 0.0 | 0.0 | 0.0 | 0.0 | 0.0 | 0.0 | 0.0 |
| 18A | 0.0 | 0.0 | 0.0 | 0.0 | 0.0 | 0.1 | 0.0 | 0.1 | 0.0 | 0.0 |
| 18B | 0.1 | 0.1 | 0.0 | 0.0 | 0.0 | 0.0 | 0.0 | 0.0 | 0.0 | 0.0 |
| 18C | 1.0 | 0.4 | 0.2 | 0.1 | 0.0 | 0.0 | 0.0 | 0.2 | 0.0 | 0.1 |
| 18F | 0.0 | 0.0 | 0.0 | 0.0 | 0.0 | 0.0 | 0.0 | 0.0 | 0.0 | 0.0 |
| 19 | 0.0 | 0.0 | 0.0 | 0.0 | 0.0 | 0.0 | 0.0 | 0.0 | 0.0 | 0.0 |
| 19A | 5.1 | 4.4 | 2.2 | 2.0 | 1.3 | 1.0 | 1.2 | 0.3 | 0.5 | 0.5 |
| 19B | 0.0 | 0.0 | 0.0 | 0.0 | 0.0 | 0.0 | 0.0 | 0.0 | 0.0 | 0.0 |
| 19C | 0.0 | 0.0 | 0.0 | 0.0 | 0.0 | 0.0 | 0.0 | 0.0 | 0.0 | 0.0 |
| 19F | 1.3 | 0.9 | 0.6 | 0.6 | 0.6 | 1.1 | 0.5 | 0.8 | 0.5 | 0.5 |
| 2 | 0.0 | 0.1 | 0.0 | 0.0 | 0.0 | 0.0 | 0.0 | 0.0 | 0.0 | 0.0 |
| 20 | 0.8 | 0.5 | 0.5 | 0.8 | 0.9 | 0.8 | 1.5 | 1.5 | 1.5 | 0.8 |
| 21 | 0.0 | 0.0 | 0.0 | 0.0 | 0.1 | 0.2 | 0.0 | 0.0 | 0.0 | 0.1 |
| 22 | 0.0 | 0.0 | 0.0 | 0.1 | 0.1 | 0.0 | 0.0 | 0.0 | 0.0 | 0.0 |
| 22A | 0.0 | 0.0 | 0.0 | 0.0 | 0.0 | 0.0 | 0.0 | 0.0 | 0.0 | 0.0 |
| 22F | 4.0 | 3.2 | 4.8 | 3.8 | 3.1 | 4.4 | 3.3 | 4.2 | 4.6 | 3.2 |
| 23 | 0.0 | 0.0 | 0.0 | 0.1 | 0.0 | 0.0 | 0.0 | 0.0 | 0.0 | 0.0 |
| 23A | 1.2 | 0.9 | 0.4 | 1.1 | 0.9 | 0.3 | 0.9 | 1.3 | 0.9 | 0.9 |
| 23B | 0.4 | 0.8 | 0.9 | 0.7 | 0.5 | 0.9 | 1.0 | 0.9 | 0.8 | 0.7 |
| 23F | 0.6 | 0.4 | 0.2 | 0.2 | 0.1 | 0.1 | 0.3 | 0.0 | 0.0 | 0.0 |
| 24 | 0.0 | 0.0 | 0.0 | 0.0 | 0.0 | 0.0 | 0.0 | 0.0 | 0.0 | 0.0 |
| 24A | 0.1 | 0.0 | 0.0 | 0.0 | 0.0 | 0.0 | 0.0 | 0.0 | 0.0 | 0.1 |
| 24B | 0.0 | 0.0 | 0.0 | 0.0 | 0.0 | 0.0 | 0.0 | 0.0 | 0.0 | 0.0 |
| 24F | 0.8 | 0.6 | 2.0 | 0.8 | 2.6 | 2.0 | 1.4 | 0.8 | 1.1 | 0.7 |
| 25 | 0.0 | 0.0 | 0.0 | 0.0 | 0.0 | 0.0 | 0.0 | 0.0 | 0.0 | 0.0 |
| 25A | 0.0 | 0.0 | 0.0 | 0.0 | 0.0 | 0.1 | 0.0 | 0.1 | 0.0 | 0.0 |
| 25F | 0.0 | 0.0 | 0.0 | 0.0 | 0.0 | 0.0 | 0.0 | 0.0 | 0.0 | 0.0 |
| 27 | 0.0 | 0.0 | 0.0 | 0.0 | 0.0 | 0.0 | 0.0 | 0.0 | 0.0 | 0.0 |
| 28 | 0.0 | 0.0 | 0.0 | 0.0 | 0.0 | 0.0 | 0.0 | 0.0 | 0.0 | 0.0 |
| 28A | 0.0 | 0.0 | 0.0 | 0.0 | 0.0 | 0.0 | 0.1 | 0.1 | 0.0 | 0.0 |
| 28F | 0.0 | 0.0 | 0.0 | 0.0 | 0.1 | 0.0 | 0.0 | 0.0 | 0.0 | 0.0 |
| 29 | 0.0 | 0.0 | 0.0 | 0.0 | 0.3 | 0.0 | 0.0 | 0.0 | 0.0 | 0.0 |
| 3 | 4.6 | 5.5 | 4.6 | 3.7 | 3.5 | 4.5 | 4.3 | 3.3 | 4.5 | 4.1 |
| 31 | 0.5 | 0.5 | 1.1 | 0.7 | 0.7 | 1.0 | 0.5 | 0.4 | 1.1 | 0.4 |
| 32 | 0.0 | 0.0 | 0.0 | 0.0 | 0.0 | 0.0 | 0.0 | 0.0 | 0.0 | 0.0 |
| 32A | 0.0 | 0.0 | 0.0 | 0.1 | 0.0 | 0.1 | 0.0 | 0.0 | 0.0 | 0.0 |
| 33 | 0.0 | 0.0 | 0.0 | 0.0 | 0.0 | 0.0 | 0.0 | 0.0 | 0.0 | 0.0 |
| 33A | 0.0 | 0.0 | 0.0 | 0.0 | 0.0 | 0.0 | 0.0 | 0.0 | 0.0 | 0.0 |
| 33B | 0.0 | 0.0 | 0.0 | 0.0 | 0.0 | 0.0 | 0.0 | 0.0 | 0.0 | 0.0 |
| 33D | 0.0 | 0.0 | 0.0 | 0.0 | 0.0 | 0.0 | 0.0 | 0.0 | 0.0 | 0.0 |
| 33F | 1.2 | 1.4 | 1.9 | 1.1 | 2.3 | 1.7 | 1.2 | 0.7 | 1.1 | 0.6 |
| 34 | 0.1 | 0.0 | 0.1 | 0.0 | 0.1 | 0.1 | 0.0 | 0.3 | 0.3 | 0.2 |
| 35 | 0.0 | 0.0 | 0.0 | 0.0 | 0.0 | 0.0 | 0.0 | 0.1 | 0.0 | 0.1 |
| 35A | 0.0 | 0.0 | 0.0 | 0.0 | 0.0 | 0.0 | 0.0 | 0.0 | 0.0 | 0.0 |
| 35B | 0.8 | 0.3 | 0.7 | 0.7 | 0.9 | 1.1 | 0.7 | 1.2 | 1.3 | 0.7 |
| 35C | 0.0 | 0.0 | 0.0 | 0.0 | 0.0 | 0.0 | 0.0 | 0.0 | 0.0 | 0.0 |
| 35F | 0.9 | 1.1 | 0.7 | 0.8 | 1.1 | 0.5 | 0.8 | 0.9 | 1.0 | 0.5 |
| 36 | 0.0 | 0.0 | 0.0 | 0.0 | 0.0 | 0.0 | 0.0 | 0.0 | 0.0 | 0.0 |
| 37 | 0.1 | 0.0 | 0.0 | 0.0 | 0.0 | 0.0 | 0.0 | 0.1 | 0.1 | 0.2 |
| 38 | 0.3 | 0.6 | 1.1 | 0.5 | 0.4 | 0.3 | 0.7 | 0.3 | 0.6 | 0.3 |
| 39 | 0.0 | 0.0 | 0.0 | 0.0 | 0.0 | 0.0 | 0.0 | 0.0 | 0.0 | 0.0 |
| 4 | 2.2 | 1.3 | 1.1 | 0.7 | 1.0 | 0.4 | 0.3 | 0.0 | 0.4 | 0.3 |
| 40 | 0.0 | 0.0 | 0.0 | 0.0 | 0.0 | 0.0 | 0.0 | 0.0 | 0.0 | 0.0 |
| 41A | 0.0 | 0.0 | 0.0 | 0.0 | 0.0 | 0.0 | 0.0 | 0.0 | 0.0 | 0.0 |
| 42 | 0.0 | 0.0 | 0.0 | 0.0 | 0.0 | 0.0 | 0.0 | 0.0 | 0.0 | 0.0 |
| 43 | 0.0 | 0.0 | 0.0 | 0.0 | 0.0 | 0.0 | 0.0 | 0.0 | 0.0 | 0.0 |
| 45 | 0.0 | 0.0 | 0.0 | 0.0 | 0.0 | 0.0 | 0.0 | 0.0 | 0.0 | 0.0 |
| 46 | 0.0 | 0.0 | 0.0 | 0.0 | 0.0 | 0.0 | 0.0 | 0.0 | 0.0 | 0.0 |
| 47F | 0.0 | 0.0 | 0.0 | 0.0 | 0.0 | 0.0 | 0.0 | 0.0 | 0.0 | 0.0 |
| 48 | 0.0 | 0.0 | 0.0 | 0.0 | 0.0 | 0.0 | 0.0 | 0.0 | 0.0 | 0.0 |
| 5 | 0.1 | 0.1 | 0.0 | 0.0 | 0.0 | 0.0 | 0.0 | 0.0 | 0.0 | 0.0 |
| 6 | 0.0 | 0.0 | 0.0 | 0.1 | 0.0 | 0.0 | 0.0 | 0.0 | 0.0 | 0.0 |
| 6A | 0.8 | 0.6 | 0.0 | 0.2 | 0.2 | 0.0 | 0.0 | 0.0 | 0.0 | 0.0 |
| 6B | 0.5 | 0.5 | 0.3 | 0.0 | 0.4 | 0.1 | 0.3 | 0.0 | 0.0 | 0.0 |
| 6C | 1.6 | 2.4 | 1.8 | 0.7 | 0.7 | 1.2 | 0.8 | 0.7 | 0.4 | 0.4 |
| 6D | 0.0 | 0.0 | 0.0 | 0.0 | 0.0 | 0.0 | 0.0 | 0.0 | 0.0 | 0.0 |
| 7 | 0.0 | 0.0 | 0.0 | 0.0 | 0.0 | 0.0 | 0.0 | 0.0 | 0.0 | 0.0 |
| 7A | 0.0 | 0.0 | 0.0 | 0.0 | 0.0 | 0.0 | 0.0 | 0.0 | 0.0 | 0.0 |
| 7B | 0.0 | 0.0 | 0.0 | 0.0 | 0.0 | 0.0 | 0.0 | 0.1 | 0.0 | 0.1 |
| 7C | 0.1 | 0.0 | 0.0 | 0.0 | 0.0 | 0.0 | 0.1 | 0.1 | 0.4 | 0.5 |
| 7F | 4.5 | 3.5 | 3.5 | 2.1 | 2.8 | 1.8 | 0.6 | 0.4 | 0.4 | 0.2 |
| 8 | 3.3 | 3.8 | 3.2 | 5.0 | 7.1 | 10.5 | 9.1 | 10.2 | 10.5 | 8.7 |
| 9 | 0.0 | 0.0 | 0.0 | 0.0 | 0.0 | 0.0 | 0.0 | 0.0 | 0.0 | 0.0 |
| 9A | 0.0 | 0.0 | 0.1 | 0.0 | 0.0 | 0.0 | 0.0 | 0.0 | 0.0 | 0.0 |
| 9L | 0.0 | 0.0 | 0.0 | 0.0 | 0.0 | 0.0 | 0.0 | 0.0 | 0.0 | 0.0 |
| 9N | 1.8 | 2.2 | 3.0 | 2.5 | 2.8 | 3.1 | 2.8 | 3.3 | 3.9 | 1.8 |
| 9V | 0.9 | 0.2 | 0.2 | 0.0 | 0.1 | 0.2 | 0.1 | 0.1 | 0.0 | 0.0 |
| **Finland** | **2010** | **2011** | **2012** | **2013** | **2014** | **2015** | **2016** | **2017** | **2018** | **2019** |
| **Serotype** |  | | | | | | | | | |
| **Total** | **32.3** | **30.4** | **33.7** | **30.2** | **32.5** | **38.0** | **36.1** | **37.2** | **32.5** | **31.4** |
| 1 | 0.0 | 0.0 | 0.0 | 0.0 | 0.1 | 0.1 | 0.0 | 0.0 | 0.0 | 0.0 |
| 10 | 0.2 | 0.0 | 0.0 | 0.2 | 0.0 | 0.1 | 0.0 | 0.0 | 0.0 | 0.0 |
| 10A | 0.0 | 0.2 | 0.2 | 0.1 | 0.3 | 0.3 | 0.2 | 0.2 | 0.5 | 0.2 |
| 10B | 0.0 | 0.0 | 0.0 | 0.0 | 0.0 | 0.1 | 0.0 | 0.0 | 0.0 | 0.0 |
| 10C | 0.0 | 0.0 | 0.0 | 0.0 | 0.0 | 0.0 | 0.0 | 0.0 | 0.0 | 0.0 |
| 10F | 0.0 | 0.0 | 0.0 | 0.0 | 0.0 | 0.0 | 0.0 | 0.1 | 0.0 | 0.0 |
| 11 | 0.0 | 0.0 | 0.0 | 0.0 | 0.0 | 0.0 | 0.0 | 0.0 | 0.0 | 0.0 |
| 11A | 0.7 | 0.9 | 1.3 | 1.4 | 0.8 | 1.6 | 0.7 | 1.0 | 1.1 | 1.1 |
| 11B | 0.1 | 0.0 | 0.0 | 0.1 | 0.0 | 0.0 | 0.1 | 0.1 | 0.0 | 0.0 |
| 11C | 0.0 | 0.1 | 0.1 | 0.0 | 0.0 | 0.0 | 0.0 | 0.0 | 0.0 | 0.0 |
| 11D | 0.0 | 0.0 | 0.0 | 0.0 | 0.0 | 0.0 | 0.0 | 0.0 | 0.0 | 0.0 |
| 11E | 0.0 | 0.0 | 0.0 | 0.0 | 0.0 | 0.0 | 0.0 | 0.0 | 0.0 | 0.0 |
| 11F | 0.0 | 0.0 | 0.0 | 0.0 | 0.0 | 0.0 | 0.0 | 0.0 | 0.0 | 0.0 |
| 12 | 0.0 | 0.0 | 0.0 | 0.0 | 0.0 | 0.0 | 0.0 | 0.0 | 0.0 | 0.0 |
| 12A | 0.0 | 0.0 | 0.0 | 0.0 | 0.0 | 0.0 | 0.0 | 0.0 | 0.0 | 0.0 |
| 12B | 0.0 | 0.0 | 0.0 | 0.0 | 0.0 | 0.0 | 0.0 | 0.0 | 0.0 | 0.0 |
| 12F | 0.0 | 0.0 | 0.1 | 0.1 | 0.3 | 0.2 | 0.1 | 0.1 | 0.0 | 0.2 |
| 13 | 0.0 | 0.0 | 0.0 | 0.0 | 0.2 | 0.0 | 0.0 | 0.3 | 0.1 | 0.0 |
| 14 | 5.2 | 4.7 | 4.8 | 2.8 | 2.1 | 0.4 | 0.7 | 0.9 | 0.5 | 0.1 |
| 15 | 0.0 | 0.0 | 0.0 | 0.0 | 0.1 | 0.0 | 0.0 | 0.0 | 0.0 | 0.0 |
| 15A | 0.2 | 0.2 | 0.3 | 0.3 | 0.2 | 0.5 | 1.0 | 0.4 | 0.4 | 1.1 |
| 15B | 0.0 | 0.4 | 0.5 | 0.6 | 0.9 | 0.7 | 0.8 | 0.4 | 0.4 | 1.0 |
| 15B/C | 0.0 | 0.0 | 0.0 | 0.0 | 0.0 | 0.0 | 0.0 | 0.0 | 0.0 | 0.0 |
| 15C | 0.2 | 0.2 | 0.5 | 0.2 | 0.3 | 0.5 | 0.9 | 0.3 | 0.4 | 0.5 |
| 15F | 0.0 | 0.0 | 0.0 | 0.0 | 0.0 | 0.0 | 0.0 | 0.0 | 0.0 | 0.0 |
| 16 | 0.0 | 0.0 | 0.0 | 0.0 | 0.0 | 0.0 | 0.0 | 0.0 | 0.0 | 0.0 |
| 16A | 0.0 | 0.0 | 0.0 | 0.0 | 0.0 | 0.0 | 0.0 | 0.0 | 0.0 | 0.0 |
| 16F | 0.2 | 0.2 | 0.3 | 0.1 | 0.0 | 0.4 | 0.4 | 0.2 | 0.3 | 0.2 |
| 17 | 0.0 | 0.2 | 0.1 | 0.0 | 0.0 | 0.0 | 0.0 | 0.0 | 0.0 | 0.0 |
| 17A | 0.0 | 0.0 | 0.0 | 0.0 | 0.0 | 0.0 | 0.0 | 0.0 | 0.0 | 0.0 |
| 17F | 0.0 | 0.0 | 0.0 | 0.1 | 0.0 | 0.0 | 0.0 | 0.3 | 0.3 | 0.2 |
| 18 | 0.0 | 0.0 | 0.0 | 0.0 | 0.0 | 0.0 | 0.0 | 0.0 | 0.0 | 0.0 |
| 18A | 0.0 | 0.0 | 0.0 | 0.0 | 0.0 | 0.0 | 0.0 | 0.0 | 0.0 | 0.0 |
| 18B | 0.1 | 0.0 | 0.0 | 0.0 | 0.0 | 0.0 | 0.0 | 0.0 | 0.0 | 0.0 |
| 18C | 0.9 | 1.3 | 0.9 | 0.8 | 0.5 | 1.0 | 0.2 | 0.4 | 0.3 | 0.1 |
| 18F | 0.0 | 0.0 | 0.0 | 0.0 | 0.0 | 0.0 | 0.1 | 0.0 | 0.0 | 0.0 |
| 19 | 0.0 | 0.0 | 0.0 | 0.0 | 0.0 | 0.0 | 0.0 | 0.0 | 0.0 | 0.0 |
| 19A | 1.1 | 1.7 | 2.1 | 3.3 | 3.7 | 5.5 | 6.9 | 8.5 | 7.1 | 6.6 |
| 19B | 0.0 | 0.0 | 0.0 | 0.0 | 0.0 | 0.0 | 0.0 | 0.0 | 0.0 | 0.0 |
| 19C | 0.0 | 0.0 | 0.0 | 0.0 | 0.0 | 0.0 | 0.0 | 0.0 | 0.0 | 0.0 |
| 19F | 2.3 | 0.9 | 1.5 | 0.8 | 0.5 | 0.6 | 0.8 | 0.1 | 0.4 | 0.6 |
| 2 | 0.0 | 0.0 | 0.0 | 0.0 | 0.0 | 0.0 | 0.0 | 0.0 | 0.0 | 0.0 |
| 20 | 0.0 | 0.1 | 0.1 | 0.0 | 0.3 | 0.0 | 0.1 | 0.2 | 0.3 | 0.1 |
| 21 | 0.0 | 0.0 | 0.0 | 0.0 | 0.0 | 0.0 | 0.0 | 0.0 | 0.0 | 0.0 |
| 22 | 0.0 | 0.0 | 0.0 | 0.0 | 0.0 | 0.0 | 0.0 | 0.0 | 0.0 | 0.0 |
| 22A | 0.0 | 0.0 | 0.0 | 0.0 | 0.0 | 0.0 | 0.0 | 0.0 | 0.0 | 0.1 |
| 22F | 2.5 | 2.8 | 2.7 | 2.7 | 3.9 | 4.0 | 4.2 | 3.4 | 2.7 | 3.6 |
| 23 | 0.0 | 0.0 | 0.0 | 0.0 | 0.0 | 0.0 | 0.0 | 0.0 | 0.0 | 0.0 |
| 23A | 0.3 | 0.3 | 0.8 | 0.6 | 0.8 | 1.9 | 1.6 | 1.3 | 1.2 | 0.7 |
| 23B | 0.1 | 0.0 | 0.1 | 0.1 | 0.1 | 0.9 | 0.2 | 1.2 | 1.5 | 1.1 |
| 23F | 2.8 | 2.3 | 2.5 | 2.2 | 2.1 | 1.4 | 0.7 | 0.8 | 0.3 | 0.1 |
| 24 | 0.0 | 0.0 | 0.2 | 0.0 | 0.3 | 0.0 | 0.0 | 0.0 | 0.0 | 0.0 |
| 24A | 0.0 | 0.0 | 0.0 | 0.0 | 0.0 | 0.0 | 0.0 | 0.0 | 0.0 | 0.0 |
| 24B | 0.0 | 0.0 | 0.0 | 0.0 | 0.0 | 0.0 | 0.0 | 0.0 | 0.0 | 0.0 |
| 24F | 0.0 | 0.0 | 0.0 | 0.0 | 0.0 | 0.3 | 0.3 | 0.2 | 0.3 | 0.2 |
| 25 | 0.0 | 0.0 | 0.0 | 0.0 | 0.0 | 0.0 | 0.0 | 0.0 | 0.0 | 0.0 |
| 25A | 0.0 | 0.0 | 0.0 | 0.0 | 0.0 | 0.0 | 0.0 | 0.0 | 0.0 | 0.0 |
| 25F | 0.0 | 0.0 | 0.0 | 0.0 | 0.0 | 0.0 | 0.0 | 0.0 | 0.0 | 0.0 |
| 27 | 0.0 | 0.0 | 0.0 | 0.0 | 0.0 | 0.0 | 0.0 | 0.0 | 0.0 | 0.0 |
| 28 | 0.0 | 0.0 | 0.0 | 0.0 | 0.0 | 0.0 | 0.0 | 0.0 | 0.0 | 0.0 |
| 28A | 0.0 | 0.0 | 0.0 | 0.0 | 0.0 | 0.0 | 0.0 | 0.2 | 0.1 | 0.0 |
| 28F | 0.0 | 0.0 | 0.0 | 0.0 | 0.0 | 0.0 | 0.0 | 0.0 | 0.0 | 0.0 |
| 29 | 0.0 | 0.0 | 0.0 | 0.0 | 0.1 | 0.0 | 0.0 | 0.0 | 0.0 | 0.0 |
| 3 | 3.3 | 3.6 | 3.6 | 3.5 | 5.8 | 7.0 | 6.5 | 6.4 | 4.3 | 4.7 |
| 31 | 0.0 | 0.0 | 0.1 | 0.0 | 0.1 | 0.1 | 0.1 | 0.1 | 0.3 | 0.2 |
| 32 | 0.0 | 0.0 | 0.0 | 0.0 | 0.0 | 0.0 | 0.0 | 0.0 | 0.0 | 0.0 |
| 32A | 0.0 | 0.0 | 0.0 | 0.0 | 0.0 | 0.0 | 0.0 | 0.0 | 0.0 | 0.0 |
| 33 | 0.2 | 0.3 | 0.4 | 0.4 | 0.5 | 0.0 | 0.0 | 0.0 | 0.0 | 0.0 |
| 33A | 0.0 | 0.0 | 0.0 | 0.0 | 0.0 | 0.0 | 0.0 | 0.1 | 0.0 | 0.0 |
| 33B | 0.0 | 0.0 | 0.0 | 0.0 | 0.0 | 0.0 | 0.0 | 0.0 | 0.0 | 0.0 |
| 33D | 0.0 | 0.0 | 0.0 | 0.0 | 0.0 | 0.0 | 0.0 | 0.0 | 0.0 | 0.0 |
| 33F | 0.0 | 0.0 | 0.0 | 0.0 | 0.0 | 0.4 | 0.7 | 0.3 | 0.5 | 0.2 |
| 34 | 0.1 | 0.0 | 0.0 | 0.2 | 0.1 | 0.0 | 0.2 | 0.0 | 0.1 | 0.2 |
| 35 | 0.0 | 0.0 | 0.0 | 0.0 | 0.0 | 0.0 | 0.0 | 0.0 | 0.0 | 0.0 |
| 35A | 0.0 | 0.0 | 0.0 | 0.0 | 0.0 | 0.0 | 0.0 | 0.0 | 0.0 | 0.0 |
| 35B | 0.4 | 0.1 | 0.7 | 0.5 | 0.6 | 0.4 | 0.3 | 0.8 | 0.8 | 0.7 |
| 35C | 0.0 | 0.0 | 0.0 | 0.0 | 0.0 | 0.0 | 0.0 | 0.0 | 0.0 | 0.0 |
| 35F | 0.4 | 0.2 | 0.5 | 0.9 | 0.7 | 1.3 | 0.6 | 0.9 | 0.7 | 0.3 |
| 36 | 0.0 | 0.0 | 0.0 | 0.0 | 0.0 | 0.0 | 0.0 | 0.0 | 0.0 | 0.0 |
| 37 | 0.0 | 0.0 | 0.0 | 0.0 | 0.0 | 0.0 | 0.0 | 0.0 | 0.1 | 0.0 |
| 38 | 0.0 | 0.0 | 0.2 | 0.2 | 0.3 | 0.1 | 0.1 | 0.2 | 0.0 | 0.2 |
| 39 | 0.0 | 0.0 | 0.0 | 0.0 | 0.0 | 0.0 | 0.0 | 0.0 | 0.0 | 0.0 |
| 4 | 2.1 | 1.6 | 1.5 | 1.8 | 1.1 | 1.3 | 1.2 | 0.9 | 1.1 | 0.3 |
| 40 | 0.0 | 0.0 | 0.0 | 0.1 | 0.0 | 0.1 | 0.0 | 0.0 | 0.0 | 0.0 |
| 41A | 0.0 | 0.0 | 0.0 | 0.0 | 0.0 | 0.0 | 0.0 | 0.0 | 0.0 | 0.0 |
| 42 | 0.0 | 0.0 | 0.0 | 0.0 | 0.0 | 0.0 | 0.0 | 0.0 | 0.0 | 0.0 |
| 43 | 0.0 | 0.0 | 0.0 | 0.0 | 0.0 | 0.0 | 0.0 | 0.0 | 0.0 | 0.0 |
| 45 | 0.0 | 0.0 | 0.0 | 0.0 | 0.0 | 0.0 | 0.0 | 0.0 | 0.0 | 0.0 |
| 46 | 0.0 | 0.0 | 0.0 | 0.0 | 0.0 | 0.0 | 0.0 | 0.0 | 0.0 | 0.0 |
| 47F | 0.0 | 0.0 | 0.0 | 0.0 | 0.0 | 0.0 | 0.0 | 0.0 | 0.0 | 0.0 |
| 48 | 0.0 | 0.0 | 0.0 | 0.0 | 0.0 | 0.0 | 0.0 | 0.0 | 0.0 | 0.0 |
| 5 | 0.1 | 0.0 | 0.0 | 0.0 | 0.0 | 0.0 | 0.0 | 0.0 | 0.0 | 0.0 |
| 6 | 0.0 | 0.0 | 0.0 | 0.0 | 0.0 | 0.0 | 0.0 | 0.0 | 0.0 | 0.0 |
| 6A | 1.4 | 1.1 | 0.6 | 0.9 | 1.7 | 1.1 | 0.9 | 0.9 | 0.7 | 0.2 |
| 6B | 2.7 | 2.0 | 1.6 | 0.9 | 0.5 | 1.2 | 0.4 | 0.5 | 0.4 | 0.2 |
| 6C | 0.1 | 0.7 | 1.5 | 1.2 | 0.7 | 2.7 | 2.8 | 3.8 | 3.4 | 4.2 |
| 6D | 0.2 | 0.0 | 0.0 | 0.1 | 0.0 | 0.0 | 0.1 | 0.0 | 0.0 | 0.0 |
| 7 | 0.0 | 0.0 | 0.0 | 0.0 | 0.0 | 0.0 | 0.0 | 0.0 | 0.0 | 0.0 |
| 7A | 0.0 | 0.0 | 0.0 | 0.0 | 0.0 | 0.0 | 0.0 | 0.0 | 0.0 | 0.0 |
| 7B | 0.0 | 0.0 | 0.0 | 0.0 | 0.0 | 0.0 | 0.0 | 0.0 | 0.0 | 0.0 |
| 7C | 0.0 | 0.1 | 0.1 | 0.1 | 0.0 | 0.0 | 0.0 | 0.0 | 0.0 | 0.2 |
| 7F | 1.6 | 1.6 | 1.1 | 0.8 | 0.8 | 0.5 | 0.3 | 0.1 | 0.4 | 0.0 |
| 8 | 0.0 | 0.2 | 0.3 | 0.1 | 0.4 | 0.1 | 1.0 | 0.9 | 0.6 | 1.0 |
| 9 | 0.0 | 0.0 | 0.0 | 0.0 | 0.0 | 0.0 | 0.0 | 0.0 | 0.0 | 0.0 |
| 9A | 0.0 | 0.0 | 0.0 | 0.0 | 0.0 | 0.0 | 0.0 | 0.0 | 0.0 | 0.0 |
| 9L | 0.0 | 0.0 | 0.0 | 0.0 | 0.0 | 0.0 | 0.0 | 0.0 | 0.0 | 0.0 |
| 9N | 1.7 | 1.2 | 1.5 | 1.5 | 0.9 | 1.0 | 0.6 | 0.9 | 0.6 | 0.8 |
| 9V | 0.8 | 1.1 | 0.9 | 0.8 | 0.9 | 0.1 | 0.5 | 0.3 | 0.3 | 0.2 |
| **Norway** | **2010** | **2011** | **2012** | **2013** | **2014** | **2015** | **2016** | **2017** | **2018** | **2019** |
| **Serotype** |  | | | | | | | | | |
| **Total** | **54.0** | **51.5** | **42.6** | **38.8** | **38.3** | **37.4** | **44.1** | **37.8** | **38.5** | **38.1** |
| 1 | 2.0 | 2.1 | 0.9 | 0.4 | 0.5 | 0.0 | 0.0 | 0.0 | 0.0 | 0.0 |
| 10 | 0.0 | 0.0 | 0.0 | 0.0 | 0.0 | 0.0 | 0.0 | 0.0 | 0.0 | 0.0 |
| 10A | 1.2 | 0.5 | 0.9 | 1.2 | 1.0 | 1.2 | 1.3 | 1.3 | 2.1 | 1.4 |
| 10B | 0.0 | 0.0 | 0.0 | 0.0 | 0.3 | 0.4 | 0.4 | 0.0 | 0.0 | 0.2 |
| 10C | 0.0 | 0.0 | 0.0 | 0.0 | 0.0 | 0.0 | 0.0 | 0.1 | 0.0 | 0.0 |
| 10F | 0.0 | 0.0 | 0.0 | 0.0 | 0.1 | 0.0 | 0.1 | 0.1 | 0.2 | 0.0 |
| 11 | 0.0 | 0.0 | 0.0 | 0.0 | 0.0 | 0.0 | 0.0 | 0.0 | 0.0 | 0.0 |
| 11A | 1.9 | 1.5 | 1.6 | 1.8 | 1.8 | 1.6 | 1.2 | 1.0 | 0.6 | 1.0 |
| 11B | 0.0 | 0.0 | 0.0 | 0.0 | 0.0 | 0.0 | 0.0 | 0.0 | 0.0 | 0.2 |
| 11C | 0.0 | 0.0 | 0.0 | 0.0 | 0.0 | 0.0 | 0.0 | 0.0 | 0.0 | 0.0 |
| 11D | 0.0 | 0.0 | 0.0 | 0.0 | 0.0 | 0.0 | 0.0 | 0.0 | 0.0 | 0.0 |
| 11E | 0.0 | 0.0 | 0.0 | 0.0 | 0.0 | 0.0 | 0.0 | 0.0 | 0.0 | 0.6 |
| 11F | 0.0 | 0.0 | 0.0 | 0.0 | 0.0 | 0.0 | 0.0 | 0.0 | 0.0 | 0.0 |
| 12 | 0.0 | 0.0 | 0.0 | 0.0 | 0.0 | 0.0 | 0.0 | 0.0 | 0.0 | 0.0 |
| 12A | 0.0 | 0.0 | 0.1 | 0.0 | 0.0 | 0.0 | 0.0 | 0.0 | 0.0 | 0.0 |
| 12B | 0.0 | 0.0 | 0.0 | 0.0 | 0.0 | 0.0 | 0.0 | 0.0 | 0.0 | 0.0 |
| 12F | 0.1 | 0.5 | 0.3 | 0.5 | 0.5 | 0.4 | 1.2 | 0.5 | 0.6 | 0.0 |
| 13 | 0.0 | 0.0 | 0.0 | 0.0 | 0.1 | 0.0 | 0.2 | 0.1 | 0.0 | 0.2 |
| 14 | 1.4 | 0.7 | 0.5 | 0.4 | 0.3 | 0.2 | 0.2 | 0.1 | 0.0 | 0.4 |
| 15 | 0.0 | 0.0 | 0.0 | 0.0 | 0.0 | 0.0 | 0.0 | 0.0 | 0.4 | 0.0 |
| 15A | 0.0 | 0.1 | 0.8 | 0.5 | 1.3 | 1.1 | 1.6 | 1.9 | 1.9 | 1.4 |
| 15B | 0.9 | 1.1 | 0.9 | 0.7 | 0.8 | 0.5 | 1.1 | 0.6 | 1.3 | 0.8 |
| 15B/C | 0.0 | 0.0 | 0.0 | 0.0 | 0.0 | 0.0 | 0.0 | 0.0 | 0.0 | 0.0 |
| 15C | 0.6 | 0.5 | 0.4 | 0.1 | 1.1 | 1.0 | 0.6 | 0.7 | 0.2 | 0.8 |
| 15F | 0.0 | 0.0 | 0.0 | 0.0 | 0.0 | 0.0 | 0.0 | 0.0 | 0.0 | 0.0 |
| 16 | 0.0 | 0.0 | 0.0 | 0.0 | 0.0 | 0.0 | 0.0 | 0.0 | 0.0 | 0.0 |
| 16A | 0.0 | 0.0 | 0.0 | 0.0 | 0.1 | 0.0 | 0.0 | 0.0 | 0.0 | 0.0 |
| 16F | 1.4 | 0.5 | 0.5 | 0.9 | 0.9 | 0.7 | 1.7 | 0.7 | 1.0 | 1.2 |
| 17 | 0.0 | 0.0 | 0.0 | 0.0 | 0.0 | 0.0 | 0.0 | 0.0 | 0.0 | 0.0 |
| 17A | 0.0 | 0.0 | 0.0 | 0.0 | 0.0 | 0.0 | 0.0 | 0.0 | 0.0 | 0.0 |
| 17F | 0.1 | 0.1 | 0.3 | 0.1 | 0.1 | 0.1 | 0.1 | 0.3 | 0.6 | 0.4 |
| 18 | 0.0 | 0.0 | 0.0 | 0.0 | 0.0 | 0.0 | 0.0 | 0.0 | 0.0 | 0.0 |
| 18A | 0.3 | 0.0 | 0.0 | 0.0 | 0.1 | 0.2 | 0.0 | 0.0 | 0.0 | 0.0 |
| 18B | 0.0 | 0.0 | 0.0 | 0.0 | 0.0 | 0.0 | 0.0 | 0.0 | 0.0 | 0.0 |
| 18C | 1.0 | 0.1 | 0.3 | 0.3 | 0.4 | 0.1 | 0.0 | 0.1 | 0.0 | 0.2 |
| 18F | 0.0 | 0.0 | 0.0 | 0.0 | 0.0 | 0.0 | 0.0 | 0.0 | 0.0 | 0.0 |
| 19 | 0.0 | 0.0 | 0.0 | 0.0 | 0.0 | 0.0 | 0.0 | 0.0 | 0.0 | 0.0 |
| 19A | 6.8 | 7.0 | 3.7 | 2.0 | 2.1 | 1.5 | 1.8 | 0.8 | 1.1 | 0.4 |
| 19B | 0.0 | 0.0 | 0.0 | 0.0 | 0.0 | 0.0 | 0.0 | 0.0 | 0.0 | 0.0 |
| 19C | 0.0 | 0.0 | 0.0 | 0.0 | 0.0 | 0.0 | 0.0 | 0.0 | 0.0 | 0.0 |
| 19F | 1.2 | 1.1 | 0.8 | 0.4 | 0.3 | 0.5 | 0.6 | 0.3 | 0.4 | 0.8 |
| 2 | 0.0 | 0.0 | 0.0 | 0.0 | 0.0 | 0.0 | 0.0 | 0.0 | 0.0 | 0.0 |
| 20 | 0.1 | 0.0 | 0.0 | 0.1 | 0.1 | 0.0 | 0.2 | 0.7 | 0.0 | 0.8 |
| 21 | 0.1 | 0.0 | 0.1 | 0.0 | 0.3 | 0.1 | 0.0 | 0.0 | 0.2 | 0.0 |
| 22 | 0.0 | 0.0 | 0.0 | 0.0 | 0.0 | 0.0 | 0.0 | 0.0 | 0.0 | 0.0 |
| 22A | 0.0 | 0.0 | 0.0 | 0.0 | 0.0 | 0.0 | 0.1 | 0.0 | 0.0 | 0.0 |
| 22F | 6.2 | 6.5 | 6.2 | 7.7 | 6.1 | 7.8 | 9.3 | 6.6 | 7.0 | 5.5 |
| 23 | 0.0 | 0.0 | 0.0 | 0.0 | 0.0 | 0.0 | 0.0 | 0.0 | 0.0 | 0.0 |
| 23A | 2.3 | 2.2 | 1.2 | 1.6 | 2.3 | 1.7 | 0.7 | 2.1 | 1.1 | 1.6 |
| 23B | 0.3 | 1.2 | 2.0 | 2.5 | 1.5 | 1.8 | 1.0 | 1.4 | 1.5 | 2.2 |
| 23F | 0.7 | 0.8 | 0.3 | 0.4 | 0.1 | 0.0 | 0.0 | 0.2 | 0.0 | 0.0 |
| 24 | 0.0 | 0.0 | 0.0 | 0.0 | 0.0 | 0.0 | 0.0 | 0.0 | 0.0 | 0.0 |
| 24A | 0.0 | 0.0 | 0.0 | 0.1 | 0.0 | 0.0 | 0.0 | 0.0 | 0.0 | 0.0 |
| 24B | 0.0 | 0.0 | 0.0 | 0.0 | 0.0 | 0.0 | 0.0 | 0.0 | 0.0 | 0.0 |
| 24F | 0.1 | 0.1 | 0.3 | 0.3 | 0.8 | 1.7 | 2.2 | 1.4 | 0.2 | 1.0 |
| 25 | 0.0 | 0.0 | 0.0 | 0.0 | 0.0 | 0.0 | 0.0 | 0.0 | 0.0 | 0.0 |
| 25A | 0.0 | 0.0 | 0.0 | 0.0 | 0.0 | 0.0 | 0.0 | 0.0 | 0.0 | 0.0 |
| 25F | 0.0 | 0.0 | 0.0 | 0.0 | 0.0 | 0.0 | 0.1 | 0.0 | 0.0 | 0.0 |
| 27 | 0.0 | 0.0 | 0.0 | 0.0 | 0.0 | 0.0 | 0.0 | 0.0 | 0.0 | 0.0 |
| 28 | 0.0 | 0.0 | 0.0 | 0.0 | 0.0 | 0.0 | 0.0 | 0.0 | 0.0 | 0.0 |
| 28A | 0.0 | 0.0 | 0.0 | 0.0 | 0.0 | 0.0 | 0.0 | 0.0 | 0.0 | 0.0 |
| 28F | 0.1 | 0.0 | 0.0 | 0.0 | 0.0 | 0.0 | 0.0 | 0.0 | 0.0 | 0.0 |
| 29 | 0.0 | 0.0 | 0.0 | 0.0 | 0.1 | 0.0 | 0.1 | 0.1 | 0.0 | 0.0 |
| 3 | 4.2 | 3.4 | 4.0 | 2.6 | 2.6 | 2.7 | 3.9 | 5.9 | 4.8 | 5.1 |
| 31 | 0.3 | 0.0 | 0.0 | 0.3 | 0.0 | 0.7 | 1.2 | 0.6 | 0.4 | 0.2 |
| 32 | 0.0 | 0.0 | 0.0 | 0.0 | 0.0 | 0.0 | 0.0 | 0.0 | 0.0 | 0.0 |
| 32A | 0.0 | 0.0 | 0.0 | 0.0 | 0.0 | 0.0 | 0.0 | 0.0 | 0.0 | 0.0 |
| 33 | 0.0 | 0.0 | 0.0 | 0.0 | 0.0 | 0.0 | 0.0 | 0.0 | 0.2 | 0.0 |
| 33A | 0.0 | 0.0 | 0.0 | 0.0 | 0.0 | 0.0 | 0.0 | 0.1 | 0.0 | 0.0 |
| 33B | 0.0 | 0.0 | 0.0 | 0.0 | 0.0 | 0.0 | 0.0 | 0.0 | 0.2 | 0.2 |
| 33D | 0.0 | 0.0 | 0.0 | 0.0 | 0.0 | 0.0 | 0.0 | 0.0 | 0.0 | 0.0 |
| 33F | 2.3 | 2.7 | 1.2 | 1.8 | 0.8 | 1.9 | 2.3 | 1.3 | 1.3 | 1.0 |
| 34 | 0.0 | 0.0 | 0.1 | 0.1 | 0.0 | 0.1 | 0.1 | 0.1 | 0.2 | 0.0 |
| 35 | 0.0 | 0.0 | 0.0 | 0.0 | 0.0 | 0.0 | 0.0 | 0.0 | 0.0 | 0.0 |
| 35A | 0.0 | 0.0 | 0.0 | 0.0 | 0.1 | 0.0 | 0.0 | 0.0 | 0.0 | 0.0 |
| 35B | 1.0 | 1.0 | 0.9 | 0.4 | 1.0 | 0.6 | 1.0 | 0.5 | 1.3 | 0.8 |
| 35C | 0.0 | 0.0 | 0.0 | 0.1 | 0.0 | 0.0 | 0.0 | 0.0 | 0.0 | 0.0 |
| 35F | 1.2 | 1.1 | 1.2 | 1.6 | 1.3 | 1.3 | 2.2 | 0.9 | 1.5 | 0.8 |
| 36 | 0.0 | 0.0 | 0.0 | 0.0 | 0.0 | 0.0 | 0.0 | 0.0 | 0.0 | 0.0 |
| 37 | 0.1 | 0.0 | 0.0 | 0.0 | 0.1 | 0.0 | 0.0 | 0.0 | 0.2 | 0.0 |
| 38 | 0.7 | 0.4 | 0.3 | 0.9 | 0.3 | 0.8 | 0.5 | 0.2 | 0.4 | 0.4 |
| 39 | 0.0 | 0.0 | 0.0 | 0.0 | 0.0 | 0.0 | 0.0 | 0.0 | 0.0 | 0.0 |
| 4 | 2.2 | 1.2 | 1.1 | 0.8 | 0.1 | 0.2 | 0.4 | 0.1 | 0.2 | 0.2 |
| 40 | 0.0 | 0.0 | 0.0 | 0.0 | 0.0 | 0.0 | 0.0 | 0.0 | 0.0 | 0.0 |
| 41A | 0.0 | 0.0 | 0.0 | 0.0 | 0.0 | 0.0 | 0.0 | 0.0 | 0.0 | 0.0 |
| 42 | 0.0 | 0.0 | 0.0 | 0.0 | 0.0 | 0.0 | 0.0 | 0.0 | 0.0 | 0.0 |
| 43 | 0.0 | 0.0 | 0.0 | 0.0 | 0.0 | 0.0 | 0.0 | 0.0 | 0.0 | 0.0 |
| 45 | 0.0 | 0.0 | 0.0 | 0.0 | 0.0 | 0.0 | 0.0 | 0.0 | 0.0 | 0.0 |
| 46 | 0.0 | 0.0 | 0.0 | 0.0 | 0.0 | 0.0 | 0.0 | 0.0 | 0.0 | 0.0 |
| 47F | 0.0 | 0.0 | 0.0 | 0.0 | 0.0 | 0.0 | 0.0 | 0.0 | 0.0 | 0.0 |
| 48 | 0.0 | 0.0 | 0.0 | 0.0 | 0.0 | 0.0 | 0.0 | 0.0 | 0.0 | 0.0 |
| 5 | 0.0 | 0.0 | 0.0 | 0.0 | 0.0 | 0.0 | 0.0 | 0.1 | 0.0 | 0.0 |
| 6 | 0.0 | 0.0 | 0.0 | 0.0 | 0.0 | 0.0 | 0.0 | 0.0 | 0.0 | 0.0 |
| 6A | 1.4 | 1.0 | 0.7 | 0.3 | 0.1 | 0.2 | 0.1 | 0.1 | 0.2 | 0.0 |
| 6B | 0.0 | 1.1 | 0.7 | 0.4 | 0.5 | 0.1 | 0.0 | 0.2 | 0.4 | 0.0 |
| 6C | 3.6 | 4.0 | 2.7 | 2.5 | 1.5 | 1.5 | 1.0 | 0.3 | 1.7 | 1.0 |
| 6D | 0.0 | 0.0 | 0.0 | 0.0 | 0.0 | 0.0 | 0.0 | 0.0 | 0.0 | 0.0 |
| 7 | 0.0 | 0.0 | 0.0 | 0.0 | 0.0 | 0.0 | 0.0 | 0.0 | 0.0 | 0.0 |
| 7A | 0.0 | 0.0 | 0.0 | 0.0 | 0.0 | 0.0 | 0.0 | 0.0 | 0.0 | 0.0 |
| 7B | 0.0 | 0.0 | 0.0 | 0.0 | 0.1 | 0.0 | 0.0 | 0.0 | 0.0 | 0.0 |
| 7C | 0.0 | 0.0 | 0.1 | 0.0 | 0.0 | 0.0 | 0.1 | 0.1 | 0.0 | 0.2 |
| 7F | 5.3 | 5.4 | 4.7 | 3.3 | 3.1 | 1.7 | 1.7 | 0.3 | 0.4 | 0.2 |
| 8 | 1.2 | 1.2 | 1.1 | 0.9 | 1.4 | 1.2 | 2.0 | 2.3 | 2.1 | 3.3 |
| 9 | 0.0 | 0.0 | 0.0 | 0.0 | 0.0 | 0.0 | 0.0 | 0.0 | 0.0 | 0.0 |
| 9A | 0.0 | 0.0 | 0.0 | 0.0 | 0.0 | 0.0 | 0.0 | 0.0 | 0.0 | 0.0 |
| 9L | 0.0 | 0.0 | 0.0 | 0.0 | 0.0 | 0.0 | 0.0 | 0.0 | 0.2 | 0.0 |
| 9N | 0.6 | 1.2 | 1.1 | 0.8 | 1.9 | 1.5 | 1.6 | 3.1 | 2.5 | 3.9 |
| 9V | 1.0 | 0.8 | 0.5 | 0.0 | 0.4 | 0.1 | 0.2 | 0.1 | 0.0 | 0.0 |
| **Sweden** | **2010** | **2011** | **2012** | **2013** | **2014** | **2015** | **2016** | **2017** | **2018** | **2019** |
| **Serotype** |  | | | | | | | | | |
| **Total** | **47.0** | **41.1** | **44.3** | **41.6** | **38.6** | **41.1** | **43.7** | **42.9** | **41.9** | **41.8** |
| 1 | N/A | 0.2 | 0.5 | 0.2 | 0.1 | 0.1 | 0.1 | 0.1 | 0.0 | 0.1 |
| 10 | N/A | 0.0 | 0.1 | 0.0 | 0.0 | 0.0 | 0.0 | 0.0 | 0.0 | 0.0 |
| 10A | N/A | 0.7 | 0.8 | 0.9 | 1.1 | 1.8 | 1.3 | 1.1 | 1.0 | 0.9 |
| 10B | N/A | 0.0 | 0.0 | 0.1 | 0.1 | 0.1 | 0.0 | 0.1 | 0.0 | 0.1 |
| 10C | N/A | 0.0 | 0.0 | 0.0 | 0.0 | 0.0 | 0.0 | 0.0 | 0.0 | 0.0 |
| 10F | N/A | 0.0 | 0.0 | 0.1 | 0.0 | 0.1 | 0.0 | 0.0 | 0.0 | 0.1 |
| 11 | N/A | 0.0 | 0.1 | 0.0 | 0.0 | 0.0 | 0.0 | 0.0 | 0.0 | 0.0 |
| 11A | N/A | 1.1 | 1.8 | 2.0 | 1.2 | 1.5 | 1.5 | 1.5 | 0.8 | 1.7 |
| 11B | N/A | 0.0 | 0.0 | 0.0 | 0.1 | 0.0 | 0.1 | 0.0 | 0.0 | 0.0 |
| 11C | N/A | 0.0 | 0.0 | 0.0 | 0.0 | 0.0 | 0.0 | 0.0 | 0.1 | 0.0 |
| 11D | N/A | 0.0 | 0.0 | 0.0 | 0.0 | 0.0 | 0.0 | 0.0 | 0.0 | 0.0 |
| 11E | N/A | 0.0 | 0.0 | 0.0 | 0.0 | 0.0 | 0.0 | 0.0 | 0.0 | 0.0 |
| 11F | N/A | 0.0 | 0.0 | 0.0 | 0.0 | 0.0 | 0.0 | 0.0 | 0.0 | 0.0 |
| 12 | N/A | 0.0 | 0.0 | 0.0 | 0.0 | 0.0 | 0.0 | 0.0 | 0.0 | 0.0 |
| 12A | N/A | 0.0 | 0.0 | 0.0 | 0.0 | 0.0 | 0.0 | 0.0 | 0.0 | 0.0 |
| 12B | N/A | 0.0 | 0.0 | 0.0 | 0.0 | 0.0 | 0.0 | 0.0 | 0.0 | 0.0 |
| 12F | N/A | 0.2 | 0.3 | 0.4 | 0.4 | 0.5 | 1.6 | 0.9 | 0.9 | 0.8 |
| 13 | N/A | 0.0 | 0.1 | 0.0 | 0.0 | 0.1 | 0.0 | 0.0 | 0.0 | 0.0 |
| 14 | N/A | 1.9 | 1.1 | 0.4 | 0.7 | 0.4 | 0.1 | 0.2 | 0.2 | 0.2 |
| 15 | N/A | 0.0 | 0.1 | 0.0 | 0.0 | 0.0 | 0.0 | 0.0 | 0.0 | 0.0 |
| 15A | N/A | 0.1 | 0.3 | 0.7 | 0.6 | 0.9 | 1.8 | 2.0 | 2.4 | 3.0 |
| 15B | N/A | 0.6 | 1.0 | 0.9 | 0.5 | 0.6 | 0.8 | 0.8 | 0.3 | 0.9 |
| 15B/C | N/A | 0.0 | 0.0 | 0.0 | 0.0 | 0.0 | 0.0 | 0.0 | 0.0 | 0.0 |
| 15C | N/A | 0.7 | 0.4 | 0.5 | 0.6 | 0.5 | 0.5 | 0.6 | 0.4 | 0.7 |
| 15F | N/A | 0.0 | 0.1 | 0.1 | 0.0 | 0.0 | 0.0 | 0.0 | 0.0 | 0.0 |
| 16 | N/A | 0.0 | 0.0 | 0.0 | 0.0 | 0.0 | 0.0 | 0.0 | 0.0 | 0.0 |
| 16A | N/A | 0.0 | 0.0 | 0.0 | 0.0 | 0.0 | 0.0 | 0.0 | 0.0 | 0.0 |
| 16F | N/A | 0.5 | 0.4 | 0.6 | 0.8 | 1.0 | 0.8 | 0.8 | 0.9 | 0.8 |
| 17 | N/A | 0.0 | 0.0 | 0.0 | 0.0 | 0.0 | 0.0 | 0.0 | 0.0 | 0.0 |
| 17A | N/A | 0.0 | 0.0 | 0.0 | 0.1 | 0.0 | 0.0 | 0.0 | 0.0 | 0.1 |
| 17F | N/A | 0.2 | 0.1 | 0.2 | 0.2 | 0.4 | 0.3 | 0.4 | 0.6 | 0.6 |
| 18 | N/A | 0.0 | 0.1 | 0.0 | 0.0 | 0.0 | 0.0 | 0.0 | 0.0 | 0.0 |
| 18A | N/A | 0.1 | 0.1 | 0.0 | 0.0 | 0.0 | 0.0 | 0.0 | 0.1 | 0.0 |
| 18B | N/A | 0.0 | 0.1 | 0.0 | 0.1 | 0.0 | 0.0 | 0.1 | 0.0 | 0.1 |
| 18C | N/A | 0.7 | 0.3 | 0.6 | 0.2 | 0.3 | 0.4 | 0.0 | 0.2 | 0.2 |
| 18F | N/A | 0.0 | 0.0 | 0.0 | 0.0 | 0.0 | 0.0 | 0.0 | 0.0 | 0.0 |
| 19 | N/A | 0.2 | 0.2 | 0.0 | 0.0 | 0.0 | 0.0 | 0.0 | 0.0 | 0.0 |
| 19A | N/A | 3.8 | 3.2 | 3.2 | 2.9 | 3.7 | 2.5 | 3.7 | 4.6 | 4.0 |
| 19B | N/A | 0.0 | 0.0 | 0.0 | 0.0 | 0.0 | 0.0 | 0.0 | 0.0 | 0.0 |
| 19C | N/A | 0.0 | 0.0 | 0.0 | 0.0 | 0.0 | 0.0 | 0.0 | 0.0 | 0.0 |
| 19F | N/A | 0.6 | 0.9 | 0.5 | 0.4 | 0.3 | 1.1 | 0.3 | 0.5 | 0.2 |
| 2 | N/A | 0.0 | 0.1 | 0.0 | 0.0 | 0.0 | 0.0 | 0.0 | 0.0 | 0.0 |
| 20 | N/A | 0.1 | 0.0 | 0.1 | 0.0 | 0.1 | 0.1 | 0.1 | 0.2 | 0.2 |
| 21 | N/A | 0.0 | 0.0 | 0.0 | 0.1 | 0.0 | 0.0 | 0.0 | 0.1 | 0.1 |
| 22 | N/A | 0.1 | 0.1 | 0.0 | 0.0 | 0.0 | 0.0 | 0.0 | 0.0 | 0.1 |
| 22A | N/A | 0.0 | 0.0 | 0.0 | 0.0 | 0.0 | 0.0 | 0.0 | 0.0 | 0.0 |
| 22F | N/A | 5.0 | 5.3 | 5.1 | 4.8 | 4.2 | 4.8 | 4.6 | 4.3 | 2.8 |
| 23 | N/A | 0.0 | 0.1 | 0.1 | 0.0 | 0.0 | 0.0 | 0.0 | 0.0 | 0.0 |
| 23A | N/A | 1.5 | 2.1 | 1.2 | 2.1 | 1.2 | 1.1 | 2.0 | 2.0 | 1.8 |
| 23B | N/A | 0.1 | 0.4 | 0.9 | 1.6 | 1.8 | 1.5 | 1.9 | 1.1 | 1.4 |
| 23F | N/A | 2.2 | 1.3 | 0.5 | 0.4 | 0.2 | 0.1 | 0.2 | 0.1 | 0.1 |
| 24 | N/A | 0.0 | 0.0 | 0.0 | 0.0 | 0.0 | 0.0 | 0.0 | 0.0 | 0.1 |
| 24A | N/A | 0.0 | 0.0 | 0.0 | 0.0 | 0.0 | 0.0 | 0.0 | 0.0 | 0.0 |
| 24B | N/A | 0.0 | 0.0 | 0.0 | 0.0 | 0.0 | 0.0 | 0.1 | 0.0 | 0.0 |
| 24F | N/A | 0.2 | 0.3 | 0.3 | 0.5 | 1.0 | 2.2 | 1.5 | 1.1 | 0.6 |
| 25 | N/A | 0.0 | 0.0 | 0.0 | 0.0 | 0.0 | 0.0 | 0.0 | 0.0 | 0.0 |
| 25A | N/A | 0.0 | 0.0 | 0.0 | 0.0 | 0.0 | 0.0 | 0.0 | 0.0 | 0.0 |
| 25F | N/A | 0.0 | 0.0 | 0.0 | 0.0 | 0.0 | 0.0 | 0.0 | 0.0 | 0.0 |
| 27 | N/A | 0.0 | 0.0 | 0.0 | 0.0 | 0.0 | 0.0 | 0.0 | 0.0 | 0.0 |
| 28 | N/A | 0.0 | 0.0 | 0.0 | 0.0 | 0.0 | 0.0 | 0.0 | 0.0 | 0.0 |
| 28A | N/A | 0.0 | 0.0 | 0.0 | 0.1 | 0.0 | 0.0 | 0.0 | 0.0 | 0.1 |
| 28F | N/A | 0.0 | 0.0 | 0.0 | 0.0 | 0.0 | 0.0 | 0.0 | 0.0 | 0.1 |
| 29 | N/A | 0.0 | 0.0 | 0.0 | 0.0 | 0.1 | 0.0 | 0.0 | 0.0 | 0.1 |
| 3 | N/A | 4.0 | 6.5 | 7.1 | 5.5 | 5.7 | 6.8 | 6.1 | 7.3 | 6.4 |
| 31 | N/A | 0.1 | 0.3 | 0.6 | 0.2 | 0.5 | 0.7 | 0.8 | 0.8 | 0.6 |
| 32 | N/A | 0.0 | 0.0 | 0.0 | 0.0 | 0.0 | 0.0 | 0.0 | 0.0 | 0.0 |
| 32A | N/A | 0.0 | 0.0 | 0.0 | 0.0 | 0.0 | 0.0 | 0.0 | 0.0 | 0.0 |
| 33 | N/A | 0.0 | 0.0 | 0.0 | 0.0 | 0.0 | 0.0 | 0.0 | 0.0 | 0.0 |
| 33A | N/A | 0.0 | 0.0 | 0.0 | 0.0 | 0.0 | 0.0 | 0.0 | 0.1 | 0.1 |
| 33B | N/A | 0.0 | 0.0 | 0.0 | 0.0 | 0.0 | 0.0 | 0.0 | 0.0 | 0.0 |
| 33D | N/A | 0.0 | 0.0 | 0.0 | 0.0 | 0.0 | 0.0 | 0.0 | 0.0 | 0.0 |
| 33F | N/A | 1.7 | 1.8 | 2.0 | 1.8 | 2.0 | 1.5 | 0.8 | 0.7 | 0.7 |
| 34 | N/A | 0.0 | 0.0 | 0.1 | 0.1 | 0.1 | 0.1 | 0.2 | 0.1 | 0.2 |
| 35 | N/A | 0.0 | 0.1 | 0.0 | 0.0 | 0.0 | 0.0 | 0.0 | 0.0 | 0.1 |
| 35A | N/A | 0.0 | 0.0 | 0.0 | 0.1 | 0.0 | 0.0 | 0.1 | 0.0 | 0.0 |
| 35B | N/A | 0.2 | 0.6 | 0.5 | 0.6 | 0.6 | 0.9 | 0.7 | 0.7 | 0.5 |
| 35C | N/A | 0.0 | 0.0 | 0.0 | 0.0 | 0.0 | 0.0 | 0.0 | 0.0 | 0.0 |
| 35F | N/A | 1.3 | 2.1 | 1.6 | 1.3 | 1.4 | 1.7 | 1.2 | 1.1 | 1.2 |
| 36 | N/A | 0.0 | 0.0 | 0.0 | 0.0 | 0.0 | 0.0 | 0.0 | 0.0 | 0.0 |
| 37 | N/A | 0.0 | 0.0 | 0.0 | 0.0 | 0.1 | 0.0 | 0.0 | 0.0 | 0.0 |
| 38 | N/A | 0.3 | 0.7 | 0.5 | 0.2 | 0.5 | 0.2 | 0.4 | 0.1 | 0.4 |
| 39 | N/A | 0.0 | 0.0 | 0.0 | 0.0 | 0.0 | 0.0 | 0.0 | 0.0 | 0.0 |
| 4 | N/A | 1.0 | 0.8 | 0.8 | 0.3 | 0.3 | 0.2 | 0.3 | 0.3 | 0.2 |
| 40 | N/A | 0.0 | 0.0 | 0.0 | 0.0 | 0.0 | 0.0 | 0.0 | 0.0 | 0.0 |
| 41A | N/A | 0.0 | 0.0 | 0.0 | 0.0 | 0.0 | 0.0 | 0.0 | 0.0 | 0.0 |
| 42 | N/A | 0.0 | 0.0 | 0.0 | 0.0 | 0.0 | 0.0 | 0.0 | 0.0 | 0.0 |
| 43 | N/A | 0.0 | 0.0 | 0.0 | 0.0 | 0.0 | 0.0 | 0.0 | 0.0 | 0.0 |
| 45 | N/A | 0.0 | 0.0 | 0.0 | 0.0 | 0.0 | 0.0 | 0.0 | 0.0 | 0.0 |
| 46 | N/A | 0.0 | 0.0 | 0.0 | 0.0 | 0.0 | 0.0 | 0.0 | 0.0 | 0.0 |
| 47F | N/A | 0.0 | 0.0 | 0.0 | 0.0 | 0.0 | 0.0 | 0.0 | 0.0 | 0.0 |
| 48 | N/A | 0.0 | 0.0 | 0.0 | 0.0 | 0.0 | 0.0 | 0.0 | 0.0 | 0.0 |
| 5 | N/A | 0.0 | 0.1 | 0.0 | 0.0 | 0.0 | 0.0 | 0.1 | 0.0 | 0.0 |
| 6 | N/A | 0.0 | 0.1 | 0.0 | 0.0 | 0.0 | 0.0 | 0.0 | 0.0 | 0.0 |
| 6A | N/A | 2.6 | 0.6 | 1.1 | 0.3 | 0.4 | 0.3 | 0.5 | 0.1 | 0.2 |
| 6B | N/A | 1.3 | 0.9 | 0.7 | 0.8 | 0.4 | 0.2 | 0.5 | 0.3 | 0.2 |
| 6C | N/A | 2.0 | 1.8 | 2.7 | 2.5 | 2.9 | 2.7 | 2.7 | 2.6 | 2.6 |
| 6D | N/A | 0.0 | 0.0 | 0.0 | 0.0 | 0.0 | 0.0 | 0.0 | 0.0 | 0.0 |
| 7 | N/A | 0.0 | 0.1 | 0.0 | 0.0 | 0.0 | 0.0 | 0.0 | 0.0 | 0.0 |
| 7A | N/A | 0.0 | 0.0 | 0.0 | 0.0 | 0.0 | 0.0 | 0.0 | 0.0 | 0.1 |
| 7B | N/A | 0.0 | 0.1 | 0.1 | 0.1 | 0.0 | 0.0 | 0.0 | 0.0 | 0.1 |
| 7C | N/A | 0.0 | 0.0 | 0.0 | 0.1 | 0.2 | 0.2 | 0.1 | 0.1 | 0.4 |
| 7F | N/A | 2.0 | 2.1 | 1.5 | 1.8 | 0.9 | 0.6 | 0.3 | 0.3 | 0.5 |
| 8 | N/A | 0.7 | 0.8 | 0.8 | 0.9 | 1.8 | 2.1 | 2.8 | 3.7 | 3.8 |
| 9 | N/A | 0.0 | 0.1 | 0.0 | 0.0 | 0.0 | 0.0 | 0.0 | 0.0 | 0.0 |
| 9A | N/A | 0.0 | 0.1 | 0.0 | 0.0 | 0.0 | 0.0 | 0.0 | 0.0 | 0.0 |
| 9L | N/A | 0.1 | 0.0 | 0.1 | 0.0 | 0.0 | 0.0 | 0.0 | 0.0 | 0.0 |
| 9N | N/A | 1.6 | 1.8 | 1.9 | 2.2 | 2.6 | 3.0 | 2.4 | 1.9 | 2.1 |
| 9V | N/A | 1.6 | 1.3 | 0.4 | 0.4 | 0.4 | 0.2 | 0.1 | 0.1 | 0.2 |
